# PTMoreR-enabled cross-species PTM mapping and comparative phosphoproteomics across mammals

**DOI:** 10.1016/j.crmeth.2024.100859

**Published:** 2024-09-09

**Authors:** Shisheng Wang, Yi Di, Yin Yang, Barbora Salovska, Wenxue Li, Liqiang Hu, Jiahui Yin, Wenguang Shao, Dong Zhou, Jingqiu Cheng, Dan Liu, Hao Yang, Yansheng Liu

**Affiliations:** 1 Department of Pulmonary and Critical Care Medicine, Proteomics-Metabolomics Analysis Platform, and NHC Key Lab of Transplant Engineering and Immunology, West China Hospital, Sichuan University, Chengdu 610041, China; 2 Yale Cancer Biology Institute, Yale University, West Haven, CT 06516, USA; 3 Information Research Institute, Tongji University, Shanghai 200092, China; 4 State Key Laboratory of Microbial Metabolism, School of Life Science & Biotechnology, Shanghai Jiao Tong University, Shanghai 200240, China; 5 Department of Medicine, Division of Nephrology, University of Connecticut School of Medicine, Farmington, CT 06030, USA; 6 State Key Laboratory of Respiratory Health and Multimorbidity, West China Hospital, Sichuan University, Chengdu 610041, China; 7 Department of Pharmacology, Yale University School of Medicine, New Haven, CT 06520, USA; 8 Department of Biomedical Informatics & Data Science, Yale Univeristy School of Medicine, New Haven, CT 06510, USA

**Keywords:** post-translational modification, mass spectrometry-based proteomics, bioinformatics, phosphoproteomics, motif-centric, window similarity, cross-species mapping, evolution biology, site-specific enrichment analysis, kinase-substrate annotation

## Abstract

To support PTM proteomic analysis and annotation in different species, we developed PTMoreR, a user-friendly tool that considers the surrounding amino acid sequences of PTM sites during BLAST, enabling a motif-centric analysis across species. By controlling sequence window similarity, PTMoreR can map phosphoproteomic results between any two species, perform site-level functional enrichment analysis, and generate kinase-substrate networks. We demonstrate that the majority of real P-sites in mice can be inferred from experimentally derived human P-sites with PTMoreR mapping. Furthermore, the compositions of 129 mammalian phosphoproteomes can also be predicted using PTMoreR. The method also identifies cross-species phosphorylation events that occur on proteins with an increased tendency to respond to the environmental factors. Moreover, the classic kinase motifs can be extracted across mammalian species, offering an evolutionary angle for refining current motifs. PTMoreR supports PTM proteomics in non-human species and facilitates quantitative phosphoproteomic analysis.

## Highlights

• PTMoreR enables motif-centric PTM mapping and analysis across species
• PTMoreR supports PTM site-level enrichment analysis and kinase-substrate annotations
• PTMoreR helps uncover the compositions of 129 mammalian phosphoproteomes
• PTMoreR offers functional and evolutionary insights into phosphorylation diversity

## Motivation

The concept of viewing human biology and diseases through an evolutionary lens using mammals has led to a surge in landmark omics studies. Comparative and evolutionary studies of post-translational modifications (PTMs), such as phosphorylation, across species can help us understand the contributions of these modifications to biodiversity, cell fitness, and drug mechanisms. Most site-specific PTM measurements and corresponding biological annotations have been performed in humans and mice but not in other mammalian species. We developed PTMoreR to bridge this gap by leveraging motif-centric peptide sequence mapping across species and providing analysis of non-human PTM proteomic datasets.

## Graphical abstract

**Figure:**
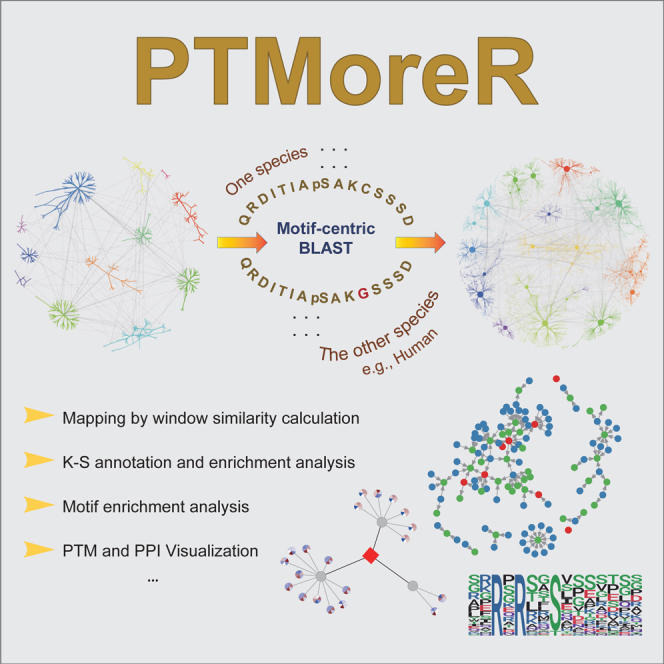

## Teaser

Wang et al. develop PTMoreR (post-translational modification ortholog aligner), a tool for mapping PTMs across species that considers the surrounding amino acid sequence of PTM sites. By extending PTM analysis to species other than humans and mice, the approach offers a valuable resource for PTM and evolution research.

## Introduction

Protein post-translational modifications (PTMs) regulate a wide range of biological activities and pathways. PTMs are normally added or removed by PTM enzymes that recognize the local structures of the substrate proteins. These structural features have been summarized as short sequence patterns around modified amino acid (aa) residues, or “motifs.” For example, representative degenerate motifs around phosphorylation sites (P-sites) have been summarized for many protein kinases,^1^^,^^2^ facilitating the functional studies of protein phosphorylation and translational studies such as cancer drug discovery. Similarly, the simplified version of sequon N-x-S/T has been recognized as the consensus motif for N-linked protein glycosylation.^3^ Due to the recent technical advances, hundreds of thousands of PTM sites have been confidently identified by mass spectrometry (MS).^4^^,^^5^^,^^6^ It is thus crucial to annotate these PTM sites, individually and on a large scale, through, for example, motif enrichment analysis.^7^^,^^8^^,^^9^

Studying PTMs such as phosphorylation across species may provide a unique understanding of drug mechanisms and aid in drug development. For example, due to the short reproduction time, high reproducibility, and cost efficiency offered by mouse-based experiments, the mouse is the most widely used animal model for human drug development. However, due to the background genomic differences between human and mouse species, a compound targeting P-site, motif, and the downstream signaling network in humans might not even exist in mice, the neglect of which will likely hamper translational medical research. However, studying qualitative and quantitative phosphorylation events across species will shed light on how phosphorylation contributes to phenotypic biodiversity, species evolution, and cell fitness. Indeed, the need to use evolutionary information to identify functional regulatory PTMs in eukaryotic proteomes has been recognized.^10^^,^^11^^,^^12^^,^^13^ A few cross-species phosphoproteomic analyses have been performed to inspect molecular mechanisms underlying exercise,^14^ proteotype co-evolution,^15^ and cancer malignancy,^16^ and for technical aspects such as the estimation of false discovery rates (FDRs) in phosphorylation databases.^17^

Despite the importance of cross-species PTM studies, to date, most site-specific PTM measurements and annotations have been performed in humans.^18^^,^^19^ Additional research efforts have been made to detect and measure PTM sites in only a few model organisms, such as mouse,^20^^,^^21^ rat,^22^^,^^23^ fly,^24^ yeast,^25^ and *Arabidopsis thaliana*,^26^ mainly due to the difficulty in establishing and studying non-human experimental models. Recent technical advances in MS, such as the combination of phosphoproteomics and data-independent acquisition MS (DIA-MS),^27^^,^^28^ have largely overcome previous idiosyncrasies in phosphoproteomic pipelines used by different laboratories, enabling reproducible profiling and accurate localization of tens of thousands of P-sites in human and mouse samples with a high throughput.^29^^,^^30^^,^^31^^,^^32^ Although real-world quantitative experiments in non-human species are definitely desirable,^15^ transferring PTM site-level knowledge to a wide range of non-human species might provide an immediate, useful resource^33^^,^^34^ and speed up the annotation and interpretation of the newly acquired PTM proteomics data from non-human species.

Mapping the orthologous PTM sites between species seems to be straightforward by using, for example, the Basic Local Alignment Search Tool (BLAST) tool available from NCBI. However, as we learned from the current motif-focused studies and the structural modeling of docking enzymes on substrates, aa residue conservation does not always imply phosphoregulatory conservation.^35^^,^^36^ For example, aa sites could be conserved between species at the residual level but differ in their “phosphorylatable” properties,^37^ due to the changes elsewhere within the motif sequence window or in the upstream signaling cascade. Therefore, it is necessary to develop a tool for PTM mapping across diverse species, with a proper consideration of motif conservation. Existing tools for PTM motif analysis are mostly single species specific or limited in their ability to compare and integrate PTM data across different species (Table S1; e.g., PTMap,^38^ PTMphinder,^39^ MoMo,^7^ MotifeR,^40^ and iPTMnet^41^). Other tools and databases such as PhosphoSitePlus,^42^ Phospho.ELM,^43^ and DAPPLE^44^ allow cross-species mapping, but rely on the known P-sites already annotated and reported in the literature. PhosphOrtholog^45^ nicely enables cross-species mapping of PTM sites, but only for four species models,^46^ and it does not directly consider motif information. In the phosphoproteomics field, PhosphoBlast,^47^ developed to compare phosphoprotein signatures among large datasets, is based on the phosphopeptide sequences but not the motif windows, and it does not support following functional annotations (Table S1). Therefore, there is a scarcity of efficient, systematic, integrative, and easy-to-use tools that are tailored for mapping and annotating PTM sites cross-species. Here, we developed a web-based and standalone software, the post-translational modification ortholog aligner, or PTMoreR, for PTM researchers and proteomic community. PTMoreR is not merely a P-site BLAST tool; instead, it considers the surrounding aa sequence of PTM sites during BLAST, enabling a motif-centric analysis across species. Additionally, PTMoreR supports a swift site-specific functional enrichment and network analysis benefiting from the well-characterized human PTM proteomic datasets. As the first validation step, we applied PTMoreR to cross-map the two most extensively measured phosphoproteomes: those from human and mouse. We found the majority of real P-sites in the mouse could be inferred from the experimentally derived human P-sites with PTMoreR mapping. We thus inferred the compositions of 129 mammalian phosphoproteomes using PTMoreR, discovering particular kinase motifs and functional features of P-sites strongly associated with mammalian evolution. We also applied PTMoreR in a phosphoproteomic dataset measured by DIA-MS for skin fibroblast cells of *Euarchontoglires* and *Laurasiatheria* species, and uncovered clade-distinctive P-sites. Finally, PTMoreR was shown to integrate SARS-CoV-2-host phosphoprotein interactions in green monkey cells. We developed a server (https://yslproteomics.shinyapps.io/PTMoreR/) that allows the user to explore results described interactively in a Shiny application.

## Results

### Overview of analysis supported by PTMoreR

**Figure 1:**
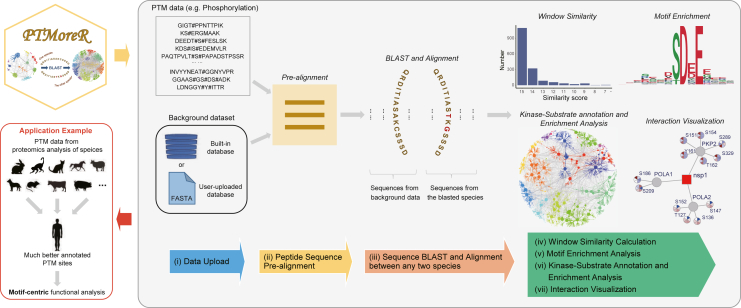
The overall workflow of PTMoreR Seven main operations were implemented: (i) data upload; (ii) peptide sequence pre-alignment; (iii) sequence BLAST and alignment between any two species; (iv) window similarity calculation; (v) motif enrichment analysis; (vi) KS annotation and enrichment analysis; and (vii) interaction visualization. See also Figure S1.

The workflow of PTMoreR is shown in Figure 1. Basically, the main aspects of the PTMoreR analysis process are as follows: (1) data upload—in this step, users should upload the identified peptide sequences, with PTMs exported directly from some common proteomic software (e.g., MaxQuant,^48^ Spectronaut^49^) or input manually prepared sequences. Users can, optionally, upload any background protein FASTA sequence database of the species used in the experiment. (2) Peptide sequence pre-alignment—after the peptide sequences with PTMs are uploaded, PTMoreR enables the alignment of peptide windows (i.e., 15-aa width by default), the retrieval of particular known motifs using user-specified regular expressions and the generation of information such as modification site positions in proteins, relevant UniProt IDs against the species they are from, using algorithms described in motifeR.^40^ (3) Sequence BLAST and alignment—PTMoreR maps protein sequences first and then finds PTM sites across different species, with default or user-defined parameters (Figure S1A). (4) Window similarity calculation—PTMoreR calculates both a simple sequence window similarity and a BLOSUM50 score^50^ between the query sequences and the blasted sequences (Figures S1B and S1C), and then allows users to set the filtering thresholds. Note that both thresholds can be at the user’s discretion and trials*.* A blasting effort between mouse, rat, and human using the PhosphoSitePlus (PSP) sites in each species and a strict match criterion suggests that 96.2%–99.6% of the proteins blasted share more than 75% of their total aa sequences, indicating a high accuracy of blast function applied to phosphoproteomics (Figures S1D–S1G). (5) Motif enrichment analysis—after the cross-species PTM site mapping, PTMoreR allows users to uncover even previously unknown motifs and helps them to discover significant PTM motifs that exist in both the query 15-mer peptides and the blasted 15-mer peptides by default. Moreover, this tool can calculate the motif position weight matrix (PWM) for users. (6) A site-specific search across mammalian species—for a given site-specific PTM based on the user’s input, this function can extract the sequences across mammalian species aligned, calculate the PWM, and evaluate and report the similarity to each PWM calculated from the substrates of every human kinase in the PhosphoSitePlus database. This function might be useful in inspecting, for example, a phosphorylation site for which the responsible kinase is not known. (7) Kinase-substrate (KS) annotation and enrichment analysis—to facilitate a site-specific (rather than a protein-specific^51^^,^^52^) functional analysis of non-human phosphoproteomic datasets, users can retrieve the KS annotation information derived from the PSP database^53^ as well as the recently established kinase library of Lewis Cantley’s group and others,^19^^,^^54^ resulting in a KS network for the query peptides and the blasted peptides, respectively. This step essentially expands the KS annotation from human to non-human species being measured. Using Fisher’s exact test, PTMoreR then infers enriched kinases based on the input of P-sites. (8) PTM and protein-protein interaction (PPI) visualization—to facilitate the analysis of the relationship between PPIs and PTMs, PTMoreR additionally supports the visualization incorporating the expression of modification sites on interacting proteins by utilizing either public or user-uploaded PPI data. Finally, all of the result tables and figures above can be downloaded.

### Estimating the functional annotation performance of PTMoreR by cross-mapping the human, mouse, and rat phosphoproteomes

**Figure 2:**
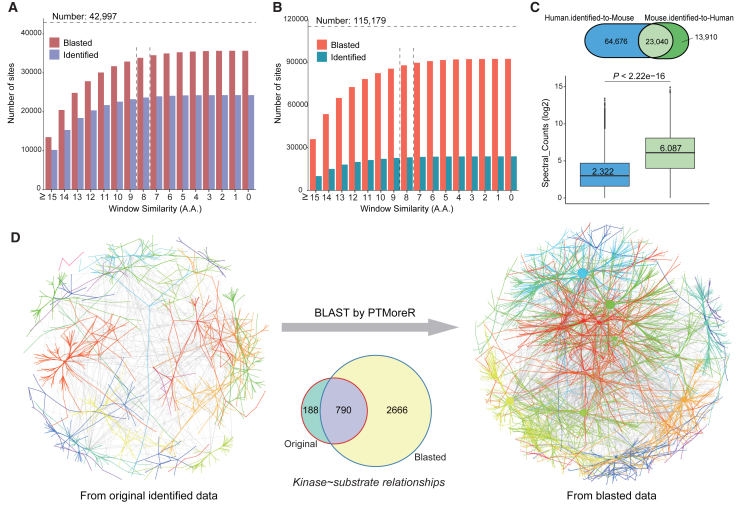
The functional annotation performance of PTMoreR by cross-mapping the human and mouse phosphoproteomes (A and B) The cumulative distribution of the mouse-to-human (A)/human-to-mouse (B) sequence window similarity scores, respectively. (C) The overlaps between the P-sites from Human.identified-to-Mouse and those from Mouse.identified-to-Human, and the boxplots of the “Spectral_Counts (log2)” shown in the Venn plot. (D) The KS annotation and network plot from the original identified data and the blasted data. The Venn plot here shows the overlaps between the KS pairs from original identified data and blasted data. See also Figures S2 and S3.

Due to its usage as the most common animal model, *Mus musculus* has an extensively measured PTM landscape among non-human species. As the first validation step assessing the general usage of PTMoreR in mapping and functionally annotating PTM sites from another species to human, we performed a cross-species analysis on mouse and human phosphoproteomes; both have been extensively measured by high-throughput MS-based phosphoproteomics. For example, two recent large-scale studies have assembled 115,179 and 42,997 P-sites for high-quality human and mouse phosphoproteomes,^18^^,^^21^ respectively, after removing duplicated and low-probability sites (marked “Human.identified” and “Mouse.identified” in Table S2). The aa distribution of Human.identified P-sites differs significantly from all theoretical S/T/Y sites, denoting the importance of MS-based phosphoproteomic identification (Figure S2A). In the first step of the mouse-to-human analysis, we treated the 42,997 mouse P-sites as input. After pre-alignment and P-site BLAST by PTMoreR, we obtained a cumulative distribution of the mouse-to-human sequence window similarity scores based on the ±7 aa (i.e., the 15-mer peptide sequence window that is used for most motif enrichment analyses) (Figure 2A). We found that, as the similarity score decreases from 15 to 0, the cumulative numbers of the phosphopeptides blasted to human sequences (labeled “Blasted”) and those identified in human phosphoproteomes (labeled “Identified”) both increased, with the cumulative hits slowing down gradually if the similarity score was below 8. Similar results were found in the human-to-mouse analysis, despite the fact that the human phosphoproteome measurement by MS has been much deeper than the mouse (Figure 2B). Aiming for a comprehensive coverage of analysis, in the present study, we took the P-sites with window similarity scores ≥8 in most analyses, a threshold used in a similar way in previous studies.^55^ To incorporate aa similarity, we also evaluated another score (i.e., BLOSUM50 scores^50^) that PTMoreR supports. The results indicated that the majority of correlation coefficients exceeded 0.85, signifying a robust relationship between the two score types (Figure S1C). Note that both score thresholds are kept open to the users and can be changed based on, for example, checking the mapping performance of PTM sites for a specific protein target. We found that using simple aa similarity scores ≥8, a total of 87,716 P-sites experimentally identified in human were successfully mapped to the corresponding sites in the mouse sequence. Intriguingly, out of these P-sites, 23,040 (62.35% of the assembled mouse dataset) were confidently identified by the comprehensive MS analysis in the mouse.^21^ (Figure 2C). Furthermore, the P-site abundance, as indicated by the spectral counts in human MS analysis,^18^ was found to be a main determining factor for the P-site to be identified in mouse measurement (*p* < 2.2e−16, Wilcoxon rank-sum test). It is therefore reasonable to expect that the majority of the 87,716 P-sites would be identified in real mouse samples if the mouse experiments could achieve a significantly deeper phosphoproteomic depth. Next, to gauge the potential annotation benefit of PTM site mapping to human, we constructed the KS network according to the results from the mouse-to-human analysis and found that the annotated KS number increased from 978 to 3,456 after PTMoreR processing (Figure 2D). Moreover, about 81% of those kinases and substrates from the originally identified phosphopeptides (790/978) were still covered, whereas 3.37 times more KS information (i.e., 2,666) was provided in the blasted results. In addition, given that the PhosphoSitePlus database has decent coverage across human, mouse, and rat phosphosites, we performed more cross-species comparisons and found similar results (Figure S3), demonstrating the reliability of PTMoreR.

### PTMoreR-based prediction of the protein phosphorylation landscape across 129 mammalian species

**Figure 3:**
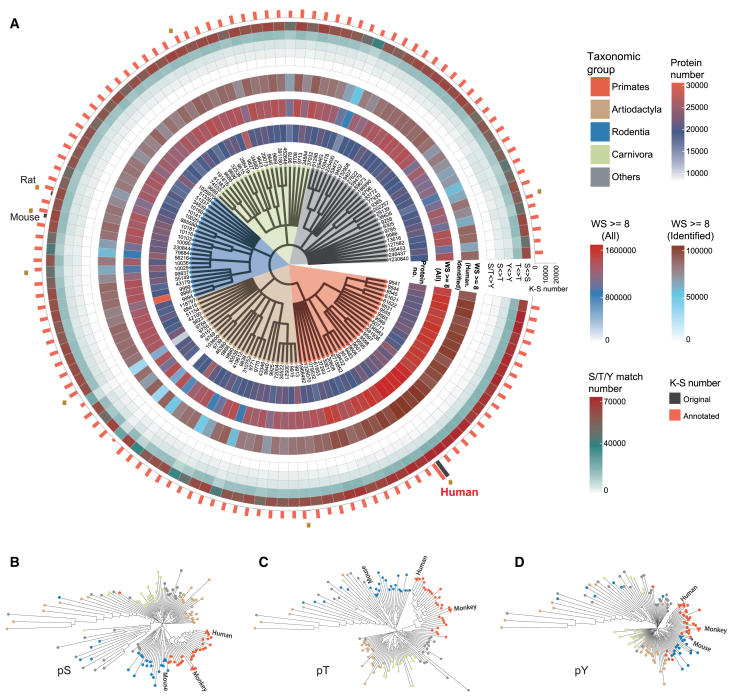
Overview of phylogenetic phosphorylation mapping atlas across 129 mammalian species (A) Circos plots visualizing the relationships (grouped into five major taxa groups), protein number, theoretical phosphopeptide number with window similarity (WS) scores above 8, identified phosphopeptide number with WS scores above 8, five types (S<>S, T<>T, Y<>Y, S<>T, and S/T<>Y) match number, and the KS annotation among the 129 mammalian species. The golden cylinders indicate that there is KS information for corresponding species in the PhosphoSitePlus database. (B–D) The clustering trees based on the sparse matrix of pS (B), pT (C), and pY (D), respectively. The node colors are the same as those of five major taxa groups. See also Figures S4 and S7.

Our above analysis indicated that substantial P-sites in a non-human mammalian species such as mouse could be inferred from experimentally derived human P-sites and PTMoreR analysis. Of note, mouse is not the phylogenetically closest mammalian to human.^56^ We thus argue that the constitutions of many other mammalian species’ phosphoproteomes could be largely inferred in a similar manner. We collected protein FASTA sequences from 129 mammalian species (including human; Table S3) and individually blasted the 128 non-human species to human protein sequences. Figure 3A discerned the resultant mapping landscape (Figure S2B). We grouped all mammalian species into five major taxa groups (Primates, Artiodactyla, Rodentia, Carnivora, and Others, in which Others comprise Chiroptera, Diprotodontia, Eulipotyphla, and Perissodactyla). The number of protein entries for the mammalian species varied from 8,000 to 31,000. The number of all theoretical S/T/Y residues with motif window similarity scores ≥8 ranged from 400,000 to 1,450,000 in these species. Next, we applied PTMoreR in a “reversed” manner (i.e., from human to other mammals) to predict the expressed and potentially detectable P-sites in each of the 128 species, resulting in 29,000–95,000 P-sites mapped to the experimentally derived human phosphoproteome (Human.identified). Due to the distinct function of tyrosine phosphorylation and the flexibility of serine and threonine residues during evolution,^57^^,^^58^ we loosened the central residue match in the identified phosphopeptides by considering and allowing five types—S<>S, T<>T, Y<>Y, S<>T, and S/T<>Y. We found that S<>S (73.08%), T<>T (19.14%), and Y<>Y (5.65%) ranked in the top three, followed by S<>T (2.01%), and S/T<>Y (0.15%). Based on the mouse and human cross-analysis above (Figure 2), most of the predicted P-sites in the 128 mammalian species mapped to Human.identified could be assumed detectable if real-world extensive MS experiments are performed in the particular species. We suggest such a prediction analysis might facilitate a global inspection of the phosphorylation conservation among mammals. Indeed, most mammalian phosphoproteomes predicted had never been experimentally analyzed or *in silico* annotated. For example, PhosphoSitePlus^53^ thus far has annotations of KS relationships for only seven non-human species (including mouse) that harbored dramatically less P-site information than human (labeled with a golden cylinder in Figure 3A); and there are no KS relationships available for the remaining 121 species. This analysis also provides a potential estimation of the conservation or diversity of all P-sites for a given protein (see epidermal growth factor receptor as an example in Figure S2C). To compare the predicted phosphoproteomic composition across species, we plotted the clustering trees based on the sparse matrix of three phosphosite types (pSer or pS, pThr or pT, and pTyr or pY in Figures 3B–3D), in which the columns were species names, the rows were identified P-sites, and the values were 0 for those sites not mapped in corresponding species and 1 for those mapped (Table S4). As expected, the clustering results illustrated that species of the five major taxonomy groups were almost clustered together, especially for primates. The P-site existence repertoire largely reflects phylogeny, and thus may contain qualitatively strong and relevant evolutionary information. The clustering tree of pY, in which Artiodactyla and Rodentia are generally clustered with Primates and Carnivora as a large cluster, exhibited a different structure than those of pS and pT, indicating a diverse evolution history between pY and pS/pT.

### Cross-species co-expression analysis revealed functional and structural diversity of P-sites associated with mammalian evolution

**Figure 4:**
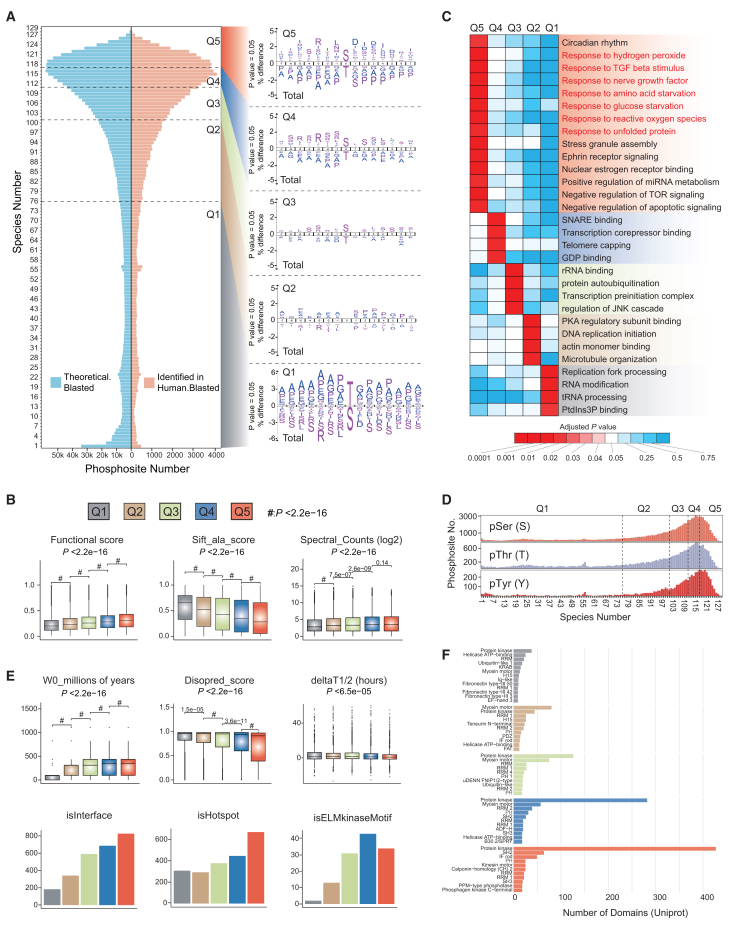
Cross-species co-expression analysis among the 129 mammalian species (A) Left: distributions of the number of the theoretical and the identified P-sites in the 129 mammals. “Blasted” means those theoretical P-sites in each species blasted to Human, and “Human.identified” means those “Blasted” P-sites identified in the identified P-sites (based on Human.identified). The WS scores here are ≥8. Right: sequence analysis of the flanking aa (±7 aa) around the pT and pS (each segment vs. total Human.identified sequences). The percentage of significant residues is shown, *p* < 0.05, *p* values, t test. (B) Distributions of the site-specific features (functional score, sift_ala_score, Spectral_Counts (log2)) of the P-sites in each segment. *p* values, Wilcoxon rank-sum test between two segments; Kruskal-Wallis rank-sum test among five segments. Error bars in boxplots are whiskers drawn within 1.5 times the interquartile range (IQR). (C) Heatmap visualizing some specific GO functions based on GO enrichment analysis of corresponding phosphoproteins in Q1–Q5, respectively. (D) Histogram of the pS, pT, and pY numbers across all species numbers (1–129). (E) Distributions of the site-specific features (W0_millions of years, disopred_score, deltaT1/2 (hours) [top] and isInterface, isHotspot, isELMkinaseMotif [bottom]) of the P-sites in each segment. *p* values, Wilcoxon rank-sum test between two segments; Kruskal-Wallis rank-sum test among five segments. Error bars in boxplots are whiskers drawn within 1.5 times the IQR. (F) Distribution of the number of P-site localized protein kinase domains based on UniProt annotation. See also Figures S4 and S6.

Next, we determined and analyzed the prevalence of P-sites across the 129 mammalian species. We first counted the number of species that exist at each P-site (Table S4) as a quantitative estimate of P-site evolution (Figure 4A). We first found that the overall distributions of species frequency were similar for all theoretical P-sites across mammalian proteomes and “identified” P-sites (based on Human.identified), underscoring the deep coverage of current human phosphoproteomics (Figure 4A, left). Next, based on the number of P-sites, we divided the species number into quintile segments, labeled Q1–Q5, with each segment encompassing a roughly equivalent number of P-sites (Figure S4A). Here, Q1 (species numbers 1–76) represents those least conserved sites, Q2–Q4 (species numbers 77–101, 102–111, 112–117, respectively) represent intermediate cases, and Q5 (species numbers 118–129) represents the most conserved sites. Within the sequence window, we compared the frequency of aa surrounding P-sites in the Q1–Q5 segments. Interestingly, we found an inversion pattern of pT and pS in the central aa position between Q1 and Q5. In particular, pS was overrepresented in Q5 and also in Q3–Q4, whereas pT was overrepresented in Q1 (Figure 4A, right). This result is suggestive of a relatively higher evolutionary conservation of pS compared to pT, which is consistent with a recent report.^59^ Furthermore, we found the aa enrichment patterns of bona fide major kinase motifs in Q4 and Q5, such as R in the −3 position, which tend to be more conserved compared to total phosphopeptide sequences. The depletion was accordingly observed in Q1. However, the surrounding A and P residues in the 15-mer window are more prevalent in Q1 and tend to be depleted in Q5, indicating the diverse distribution of these corresponding P-sites among mammals. There were no obvious preferences among Q1–Q5 segments for pY-only analysis (Figure S4B). We then confirmed that the P-site functional score^18^ significantly increases from Q1 to Q5, and the “sift_ala_score,” a computational score predicting the system tolerance if the phosphosite residue is mutated to alanine, remarkably decreases from Q1 to Q5 (Figure 4B, center), highlighting the strong positive correlation between evolutionary conservation and functional fitness^18^ (Figure 4B, left). In addition, P-sites from Q1 to Q5 were found to exhibit a small but significant increase in “Spectral_Counts (log2),” indicating the enhanced expression levels for conserved P-sites (Figure 4B, right). These results validated the statistical, functional, and evolutionary relevance of Q1–Q5 classification.

Next, to gain a functional view, we performed a Gene Ontology (GO) enrichment analysis in each of the Q1–Q5 segments based on corresponding phosphoproteins (Table S5). Specific GO functions were enriched in Q1–Q5, respectively (Figure 4C; Benjamini-Hochberg [BH]-adjusted *p* <0.01 in one of the Q1–Q5 and *p* > 0.05 in the other four). We found that many more functional items were significantly enriched in Q5, the segment embracing the most conserved P-sites. Intriguingly, in addition to circadian rhythm, which could result from the day and night cycle as a shared major environmental factor among mammalian species on Earth, a series of cellular response processes were determined to be remarkably enriched in Q5, including the responses to hydrogen peroxide, transforming growth factor β stimulus, nerve growth factor, glucose and aa starvations, and unfolded protein (Figure 4C, items highlighted in red). These results thus strongly indicate the importance of expressing P-sites and phosphoprotein orthologs to cope with a number of environmental stresses in most mammalian species. Consistently, stress granule assembly and the negative regulation of target of rapamycin and apoptotic signaling were enriched in Q5. However, processes such as replication fork processing, tRNA processing, RNA modification, and PtdIns3P binding were enriched in Q1, suggesting their strong association with mammalian phenotypic diversity, which remains to be investigated in future studies. As for the central P-site, we counted the pS, pT, and pY numbers across all species numbers and found that there is a tendency of tyrosine phosphorylation (pY) to be enriched in Q5 (Figure 4D), probably because of more conserved functions of pY than pS and pT and the role of human tyrosine residues in maintaining protein structure.^37^ To summarize, phosphoproteins of varying degrees of conservation may have distinctive functions, and the most conserved P-sites primarily enrich the response to the Earth’s environment.

We next sought to further interrogate how site-specific functional and structural features were distributed among Q1–Q5 using various scores summarized previously^18^ (Figures 4E, 4F, and S4D–S4G). As expected, the W0_millions of years (or W3_millions of years), a score denoting the age of inferred ancestral species containing the site based on the only residue (or the window of three residues), was found to have remarkably increased from Q1 to Q5 (Figures 4E, top, and S4D), indicating that many ancient P-sites are conserved across mammalian species. Also, the “Netpho_max_all” (Max Netphorest match for all models^18^^,^^36^^,^^60^) exhibited a small but significant increase (Figure S4E), which suggests that P-sites that are conserved in Q4–Q5 are a significantly better match to consensus kinase motifs than those in Q1–Q2. In addition, both the “Biological_samples” and “Pubmed_counts” gradually increased from Q1 to Q5 (Figures S4F and S4G), indicating that more conserved P-sites may be more often studied. Regarding the structural features, we found that the “disopred_score,” a computational score predicting the disordered probability of one acceptor residue, decreased from Q1 to Q5. This trend suggests that the most conserved P-sites tend to locate in the protein region with an ordered structure (Figure 4E, top). Based on site-specific discrete features,^18^ the conserved P-sites in Q4–Q5 were found to prefer to locate in protein interaction interfaces, in structural hotspots, and in eukaryotic linear kinase motifs (Figure 4E, bottom), as compared to the less conserved P-sites in Q1–Q2. According to UniProt annotation, the number of P-sites localized in protein kinase domains was also higher in Q4–Q5. A similar enrichment was observed for the SH2 domain, which was found in many proteins involved in tyrosine kinase signaling cascades (Figure 4F). Finally, we previously determined a “deltaT1/2 (hours)” value denoting the site-specific impact of phosphorylation on protein turnover using an experimental approach.^61^^,^^62^ By mapping the Q1–Q5 sites to their deltaT1/2 values, we found that Q5 P-sites harbor the shortest phosphomodiform lifetime among all sites (Figure 4E). This result agrees with our previous observation that phosphomodiforms carrying the evolutionarily important P-sites are often involved in active synthesis and degradation.^61^

In summary, PTMoreR recognized potential P-sites across mammalian species and uncovered the functional and structural features of evolutionary fundamental phosphorylation.

### PTMoreR enabled a motif-centric analysis on KSs across mammalian species

**Figure 5:**
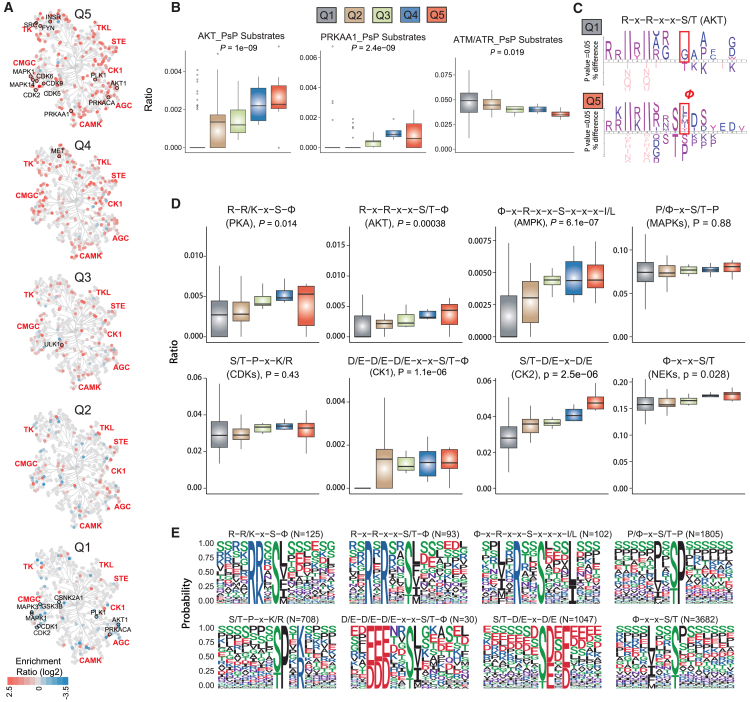
Motif-centric analysis on KSs across 129 mammalian species (A) Distribution of the kinase enrichment ratio (log2) of each segment (Q1–Q5) on the human kinase family tree. (B) Boxplots of the ratios of substrate numbers for each segment for the three kinases (AKT1, PRKAA1, ATM/ATR). Error bars in boxplots are whiskers drawn within 1.5 times the IQR. (C) Sequence analysis of the flanking aa (±7 aa) extracted according to the AKT motif of R-x-R-x-x-S/T for Q1/Q5 versus total Human.identified sequences. The percentage of significant residues is shown, *p* < 0.05, *p* values, t test. (D) Boxplots of the ratios of the eight common kinase motifs from Q1 to Q5. Error bars in boxplots are whiskers drawn within 1.5 times the IQR. (E) Motif plots of the eight common kinases in Q5.

PTMoreR itself does not assume that all kinase motifs are highly conserved through mammalian evolution. However, our analysis above may indicate that kinase motifs are closely associated with P-site conservation. We therefore asked whether this information could be used to improve our understanding of specific kinase motifs. First, based on the Q1–Q5 classification, we began by mapping each kinase in the whole kinase family tree with their known substrate P-sites annotated in the PhosphoSitePlus database^53^ (Figure 5A). This effort uncovered a total of 20 kinases showing either preferable substrate depletion or enrichment in one of the Q1–Q5 segments (BH-adjusted *p* < 0.01, Fisher’s exact test; Figure S4C; Table S6). Most of these kinases, such as CDK9, SRC, PRKACA, and AKT1, were underrepresented in Q1 and overrepresented in Q5. The ratios of substrate numbers per each Q segment were distributed for the two kinase examples, AKT1 (or AKT in general) and PRKAA1 (or AMPK), both with established motifs (Figure 5B). Notably, the ATM/ATR substrate motif was enriched in Q1, indicating that not all kinases manifest strong conservation among mammalian species.

Second, we asked whether the more conserved P-sites in Q5 are preferably coupled with specific aa for a given kinase motif. Previous studies have identified a universal AKT motif of R-x-R-x-x-S/T, whereas additional structural and peptide array studies of AKT suggested the existence of a bulky hydrophobic residue (ϕ, here referred to F, L, I, V or M^63^) at the P+1 position^29^^,^^64^ (i.e., R-x-R-x-x-S/T-ϕ). Using the regular expression-retrieving function in PTMoreR, we extracted all of the 15-mer peptides carrying R-x-R-x-x-S/T in Q1–Q5. Intriguingly, the P+1 position was found to significantly enrich the hydrophobic residues in Q5 but not Q1 and others (Figure 5C). The chances of P+1 being a hydrophobic residue in Q5 were also significantly higher than the total 15-mer peptides in Human.identified (*p* = 0.00256, Fisher’s exact test). Therefore, PTMoreR-based motif-centric analysis might help determine the consensus sequences recognized by those protein kinases conserved across species more accurately.

Third, we explored the ratio distribution of eight common motifs^1^ from Q1 to Q5 (Figures 5D and 5E), where the ratio is the quotient of the number of 15-mer peptides retrieved by PTMoreR using respective regular expressions divided by the corresponding total P-site number shared by 1–129 species (as shown in Figure 4A). Some motifs corresponding to a kinase group such as ϕ-x-x-S/T (NEKs), P/ϕ-x-S/T-P (MAPKs), and S/T-P-x-K/R (CDKs) are less specific than others. Longer motifs, such as R-x-R-x-x-S/T-ϕ (AKT), ϕ-x-R-x-x-S-x-x-x-I/L (AMPK), D/E-D/E-D/E-x-x-S/T-ϕ (CK1), and S/T-D/E-x-D/E (CK2), tended to exhibit significant patterns of increasing conservation from Q1 to Q5 (Figure 5D). Notably, the motif of P/ϕ-x-S/T-P (MAPKs) did not show a statistically different distribution among Q1 to Q5 (*p* = 0.88, Kruskal-Wallis rank-sum test), potentially implying its contribution to mammalian biodiversity. The surrounding aa of P-sites carrying these kinase motifs in Q5 were visualized and not random (Figure 5E), which may help to refine our understanding of current kinase motifs. In summary, the PTMoreR results provided an interesting and ample resource to study the kinases and their substrate motifs through an evolutionary lens.

### PTMoreR-based comparative phosphoproteomics between *Euarchontoglires* and *Laurasiatheria* clades

**Figure 6:**
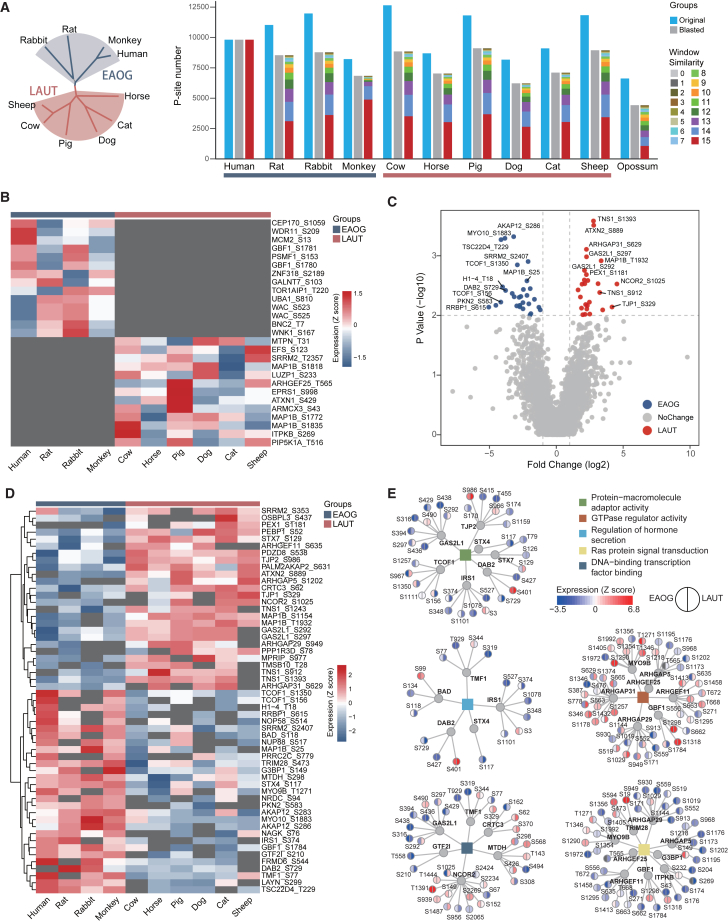
Expanded comparative phosphoproteomic analysis between *Euarchontoglires* and *Laurasiatheria* clades (A) Distributions of the number of the originally identified and the blasted P-sites across the 11 mammalian species. (B) Heatmap visualizing the P-sites that only expressed in EAOG or LAUT. (C) Volcano plots of P-site expression between EAOG and LAUT. (D) Heatmap visualizing the differentially expressed P-sites. (E) Interaction between the representative GO functions and the corresponding proteins with quantitative P-sites. See also Figure S5.

We previously reported a dataset in which there was an average of 12,400 P-sites in skin-derived fibroblast cells across 11 common mammalian species, identified by phosphoproteomic DIA-MS (or Phos-DIA).^15^ Here, we evaluated whether PTMoreR could expand this comparative phosphoproteomic analysis. Because Opossum was considered the outgroup in the original study, we focused on the other 10 species representing two major phylogenetic clades: *Euarchontoglires* (EAOG: Human, Rat, Rabbit, Monkey) and *Laurasiatheria* (LAUT: Cow, Horse, Pig, Dog, Cat, Sheep). Using PTMoreR, we extracted the unique, confidently localized P-sites and blasted these phosphopeptide sequences from non-human species to human sequences. We then kept those P-sites with window similarity scores ≥8, which resulted in a total of 26,510 union phosphopeptides across 10 mammalian species (Figures 6A and S5A). We removed those P-sites with a high missing value ratio (i.e., 40%), which was calculated using the adaptive daisy model.^65^ This resulted in 4,611 P-sites that could be compared between EAOG and LAUT groups, including missing sites in different species, greatly increasing the scope of the original phosphoproteomic data analysis, in which only ∼600 P-sites were commonly identified in all species^15^ (Table S7). Among the 4,611 P-sites, 27 were found to be expressed in either EAOG or LAUT species measured after PTMoreR mapping (Figures 6B and S5B). In addition, 26 upregulated P-sites and 29 downregulated P-sites were discovered in EAOG as compared to LAUT (fold change ≥ 2, *p* <0.01, significance analysis of microarray test^66^; Figures 6C and 6D). The GO annotation suggests that the differential P-sites between EAOG and LAUT could be annotated to biological processes, including protein-macromolecule adaptor activity, Ras protein signal transduction, DNA-binding transcription factor binding, GTPase regulator activity, regulation of hormone secretion, and focal adhesion (Figure 6E). By embracing missing data points after alignment, PTMoreR expands the quantitative phosphoproteomic analysis between species groups.

### PTMoreR visualized SARS-CoV-2-host PPIs in Vero E6 cells

**Figure 7:**
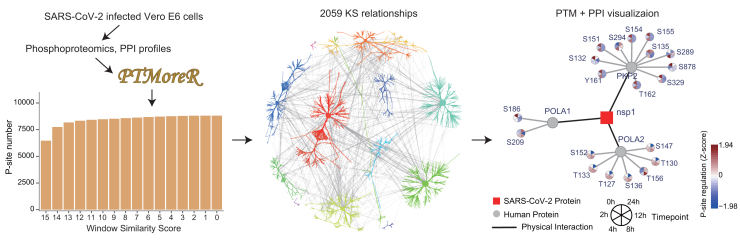
Example of analyzing the SARS-CoV-2-host PPIs in Vero E6 cells using PTMoreR The phosphoproteins/phosphopeptides were obtained from the SARS-CoV-2-infected Vero E6 cells, blasted and annotated to Human, and then visualized based on the SARS-CoV-2 virus protein-human protein interactions. See also Figure S5.

Protein PTMs sometimes facilitate PPIs, and vice versa.^67^ An additional function of PTMoreR is to visualize and integrate the PTM site-level regulation with PPIs or phosphoprotein-object relationships—for example, the object may be a common protein complex or a GO Biological Process that the phosphoproteins participate in (Figure 6E). To illustrate, we referred to another dataset in which ∼11,000 P-sites were profiled in Vero E6 cells (a green monkey cell line) infected by SARS-CoV-2 across six time points.^68^ PTMoreR mapped a total of 8,582 phosphopeptides to corresponding Human homolog sites and supported motif enrichment analysis and KS annotation in Vero E6 cells (Figures 7, S5C, and S5D). In particular, PTMoreR can visualize the PPI network (e.g., host-protein interactions) and individual P-site abundance regulation in the host proteome, similar to the analysis performed in the original report.^68^ This visualization function will allow users to explore virus-host PPIs that drive changes in phosphorylation by stereoscopic functional control over some kinases, such as Nsp1, a SARS-CoV-2 protein that disrupts mRNA decay to inhibit host gene expression (Figure 7).^69^^,^^70^

## Discussion

Previously, many translational studies had been performed in mouse models; however, not all aspects of human biology can be accurately replicated and studied in mice. Before developing specific drug compounds in mice, or before translating specific mechanistic findings from mice to humans, it is critical to confirm the consensus of the PTMs such as P-sites, as well as their corresponding motif or signaling mechanisms relevant to the drug between mice and humans. Conceivably, for studying certain diseases or developing particular drug candidates, alternative animal models such as non-human primates might have to be considered and utilized^71^^,^^72^^,^^73^; for these, PTM proteomic datasets and annotations have been unfortunately scarce so far. For example, green monkey Vero E6 cells were used as an efficient system to study the SARS-CoV-2 infection, for which in-house solutions had to be developed to map P-sites to humans in individual labs.^68^ However, the notion of seeing human biology and diseases through an evolutionary lens using mammals has recently resulted in a burst of landmark genomic studies,^74^^,^^75^^,^^76^^,^^77^^,^^78^^,^^79^ and has paved the way for the study of proteomes and PTMs in mammals in the near future.^15^^,^^80^ Indeed, PTMs play a crucial role in in variety of organisms.^81^^,^^82^^,^^83^^,^^84^ The characterization and annotation of PTMs among species could be essential for understanding fundamental evolutionary questions and more.^15^^,^^34^ To meet these needs, we herein developed a web-based, user-friendly tool, PTMoreR, to enable a motif-centric cross-species PTM analysis, with an alternative standalone version for larger datasets. The basic function of PTMoreR is simple: to map PTM sites such as phosphosites between species. However, based on this mapping function, here, we were able to compare the predicted phosphoproteomic organization across 129 mammals by using PTMoreR in a “reversed” fashion, based on the well-annotated human phosphoproteomic reference.^18^

There are a few noteworthy aspects in the design, consideration, and functions of PTMoreR. First, PTMoreR presents a motif-centric analysis. This feature also facilitates the subsequent KS mapping and network construction in PTMoreR. Using the motif extraction function, we confirmed the preferred existence of a bulky hydrophobic aa residue at the P+1 position for those most conserved AKT substrate P-sites, highlighting the potential of refining the current kinase motifs by adding evolutionary information. Second, our cross-mammal analysis is built on the relatively completed human phosphoproteome draft, which was achieved only in recent years, due to the boosted analytical performance of MS. In our analysis, we mapped 23,040 P-sites shared between human and mouse datasets, which is 33 times more than those analyzed in the early mapping studies (i.e., 700 P-sites^85^). Another relevant technical note is the improved PTM localization capacity due to the improved quality of the MS2 spectrum and the latest data acquisition schema such as Phos-DIA.^29^^,^^30^^,^^31^^,^^32^ Accordingly, site-specific PTM annotation informatic paradigms have been established.^18^^,^^21^^,^^42^ These advances warrant the feasibility of PTMoreR. Third, based on the validation of the human-to-mouse P-site mapping, we argue that the majority of undetected non-human P-sites are likely due to current MS detection limits, specific experimental conditions, and the scope of the study, rather than the sites being unphosphorylatable.^36^^,^^37^ In essence, it is plausible to translate some knowledge across species for a functional exploratory analysis even if large-scale phosphoproteomic datasets are not available for most mammals. Fourth, as shown in the EAOG vs. LAUT comparison, PTMoreR directly supports quantitative multi-species studies with larger phosphoproteomic coverage due to the tolerance of missing values. Fifth, the integrative visualization of PTM-mediated PPI^67^^,^^86^^,^^87^ in PTMoreR could be utilized for the updated protein correlation profiling datasets as well,^88^^,^^89^ offering future applications in inspecting, for example, how PTMs coordinate protein complex organization.

In our present phosphoproteomic analysis using PTMoreR mapping, we utilized two comprehensive phosphoproteomic studies, one in human and the other in mouse.^18^^,^^21^ The large PTM databases, although containing more P-sites, were not used here due to concerns about inconsistency between databases^90^ and between versions. For example, previous studies suggested that a localization probability of <90% in the databases should be filtered out to avoid significant errors during cross-species mapping.^46^ In addition, the current PTMoreR keeps the motif window similarity score open to the users, while a score ≥8 was used in the main results present. Of note, a looser window similarity^55^ may cause motifs to be missed in the target species. The similarity score is therefore recommended to be ≥14 in certain scenarios such as initial comparisons (Figures S1 and S6). The user can also use the BLOSUM50 score^50^ for motif similarity calculation, which considers the aa substitution matrix and can be useful for aligning sequences that have undergone evolutionary changes.

Our analysis has yielded a few interesting biological insights. The enrichment analysis of P-site prevalence across mammals suggests that functional PTMs are more likely to be evolutionarily conserved, and the disordered PTM regions evolve faster than the ordered regions, in agreement with the previous observations.^13^^,^^91^ Intriguingly, our results indicate that phosphorylation regulations responsible for a series of environmental traits such as day and night cycle, nutrient starvation, and oxidative stresses are conserved across mammals. Despite the recognition of environmental stress as a general evolutionary force,^92^^,^^93^ this result supports the long-term signaling adaptation of organisms in dealing with some non-extreme, regular stresses such as glucose starvation. Meanwhile, processes such as RNA modification and PtdIN3P binding were found to be fast evolving during evolution. Begik et al. revealed an unanticipated heterogeneity in the expression patterns of RNA modification-related proteins across mammalian tissues.^94^ The reason why P-sites involved in PtdIN3P binding are not conserved is not clear and might be explained by the structural roles of phosphorylation.^17^ Furthermore, P-sites whose position along the protein is a determinant of their function (positionally dependent P-sites) were found to have strong conservation,^36^ reflected by their enrichment in kinase motifs and functional domains. Finally, PTMoreR can be widely used between any species (e.g., for a given plant species mapping to Arabidopsis^95^). In addition, not limited to phosphorylation, many other modification types (e.g., N-linked protein glycosylation^3^) may also be similarly explored with PTMoreR (Figure S7).

In conclusion, we present PTMoreR, which serves as a gateway for biologists to easily retrieve PTM information for specific species of interest. It offers a valuable resource for PTM and evolution research and has the potential to provide deeper insights into the diverse roles of PTMs in cellular processes and in health and disease by adding the evolution perspective.

### Limitations of the study

There are potential limitations in our cross-species analysis. Our comparison essentially only characterizes the evolution of human P-sites, which naturally ignores the possible kinases uniquely present in other mammalian kinomes or newly emerged KS relationships that bestow a phenotypic advantage over time for a given species. Also, the human phosphoproteome datasets compiled^18^ are comprehensive, but unfortunately still not complete. For example, our recent single study applying Phos-DIA in endothelial cells^29^ identified 4,174 (i.e., 3.6%) P-sites that were not included in the reference dataset. Finally, as a general limitation in motif analysis, the existence of a particular motif does not ensure kinase docking and subsequent phosphorylation. Three-dimensional organization might be required to bring distant segments of the molecule together for phosphorylation to occur.

## Resource availability

### Lead contact

Further information and requests for resources and reagents should be directed to and will be fulfilled by Dr. Yansheng Liu (yansheng.liu@yale.edu).

### Materials availability

This study did not generate new materials.

### Data and code availability

• This paper analyzes existing, publicly available datasets. Information is also listed in the key resources table.
• The source code has been deposited at Zenodo repository and is publicly available at https://doi.org/10.5281/zenodo.10077642.
• Any additional information required to reanalyze the data reported in this paper is available from the lead contact upon request.

## Acknowledgments

We gratefully thank Drs. Benjamin E. Turk, Lilian C. Kabeche, Hongwen Zhu, and Qian Ba for their critical feedback and helpful discussions on the manuscript. Y.L. was supported by 10.13039/100005326Yale University and the US 10.13039/100000002National Institutes of Health (NIH) through grant R01GM137031, as well as a pilot grant from the 10.13039/100010900Yale Cancer Center. H.Y., S.W., and W.S. were supported by the 10.13039/501100012166National Key Research and Development Program of China (2021YFF0702003-02 to H.Y.) and the 10.13039/501100001809National Natural Science Foundation of China (32201210 to S.W.; 62102248 and 32271493 to W.S.). S.W. was also supported by the Postdoctoral Fellowship Program of CPSF of China under grant no. GZB20240490. W.S. was also supported by the 10.13039/501100012166National Key Research and Development Program of China (2022YFC3400040).

## Author contributions

Y.L. and S.W. conceived this project, designed the framework, and wrote the first version of the manuscript. S.W. performed the calculations and evaluations with guidance from Y.L. and H.Y. Y.D., Y.Y., L.H., and J.Y. helped to collect the data. Y.D., B.S., W.S., D.Z., J.C., D.L., and H.Y. contributed to the manuscript text and reviewed the manuscript. All authors approved the final manuscript. Y.L., D.L., and H.Y. supervised the study.

## Declaration of interests

The authors declare no competing interests.

## STAR★Methods

### Key resources table

**Table:** 

REAGENT or RESOURCE	SOURCE	IDENTIFIER
**Deposited data**		

Human phosphoproteome data	Ochoa et al.^18^	PRIDE database,^96^ PXD012174
Mouse phosphoproteome data	Giansanti et al.^21^	PXD030983
Phosphoproteome from ten common mammalian species	Ba et al.^15^	PXD028979
Phosphoproteome regulation in Vero E6 cells	Bouhaddou et al.^68^	PXD019113
Human glycoproteome data	N-GlycositeAtlas database^3^	http://nglycositeatlas.biomarkercenter.org
Kinase-Substrate data	PhosphoSitePlus database^53^	https://www.phosphosite.org
Kinase library data	Johnson et al.^19^ Yaron-Barir et al.^54^	https://kinase-library.mit.edu/home

**Software and algorithms**		

PTMoreR v1.0.0	This paper	https://doi.org/10.5281/zenodo.10077642
R v4.3.1	R Core Team	https://www.r-project.org
metablastr v0.3.1	Benoit et al.^97^	https://github.com/drostlab/metablastr
msa v1.32.0	Bodenhofer et al.^98^	https://bioconductor.org/packages/release/bioc/html/msa.html
ggtree v3.6.0	Xu et al.^99^	https://bioconductor.org/packages/release/bioc/html/ggtree.html
itol.toolkit v1.1.7	Zhou et al.^100^	https://cran.r-project.org/web/packages/itol.toolkit/index.html
ggseqlogo v0.1	Wagih et al.^101^	https://github.com/omarwagih/ggseqlogo
ape v5.6.2	Paradis et al.^102^	https://cran.r-project.org/web/packages/ape/index.html
pheatmap v1.0.12	Raivo Kolde	https://cran.r-project.org/web/packages/pheatmap/index.html
IceLogo	Colaert et al.^103^	https://iomics.ugent.be/icelogoserver
Coral	Metz et al.^104^	https://github.com/dphansti/CORAL
Cytoscape v3.9.1	Shannon et al.^105^	https://cytoscape.org/
samr v3.0	Li et al.^66^	https://cran.r-project.org/web/packages/samr/index.html
clusterProfiler v4.6.0	Wu et al.^106^	https://github.com/YuLab-SMU/clusterProfiler

### Method details

#### Protein sequence mapping between two species

The query protein sequences from one species were blasted to the reference proteome sequences from the other species using the blast_best_hit function with the "protein_to_protein" search type in the metablastr package.^97^ Then the best BLAST hit would be retrieved for each query protein sequence based on the following two criteria:

a. The hit with the smallest E-value which provides information about the likelihood that a given sequence match is purely by random chance and can be calculated using the following formula:

(Equation 1) $$E=m*n*2^{-S}$$

Where m means the query protein sequence length, n means the total database length (i.e., sum of all sequences), and S indicates the bit-score which measures the sequence similarity.

b. The hit with the largest matching percentage or the longest alignment length if E-values are identical. The matching percentage means the matched amino acids divided by the query protein sequence length and the alignment length means the whole sequence length after alignment.

Subsequently, the blasted pairs of sequences were aligned using the ClustalW alignment algorithm implemented in the R package msa with default parameters.^98^^,^^107^ The resulting alignments were used to convert the sequence positions of detected PTMs in the query species to positions in the reference protein sequences. These multiple sites on the same query peptide/protein are mapped to the reference peptide/protein separately. Although the sibling peptides were expected to be handled by the proteomics identification step before PTMoreR usage,^108^^,^^109^^,^^110^ if some proteins had shared peptides, *PTMoreR* will match and display all of them for users. Therefore, PTM sites and protein identifiers could be mapped to their respective reference species protein orthologs. All the functions have been integrated in *PTMoreR* and corresponding parameters are made fully open to users at their own discretion if needed (Figure S1).

#### Sequence window similarity

A sequence window here means one modified peptide with a standard width, where the modification site is in the middle position. By default, the number of left/right side characters of the central residue in *PTMoreR* is 7, which means every uploaded peptide with different lengths will be aligned into a standard window (i.e., 15 amino acids width here). For example, GIGT#PPNTTPIK to QEVKGIGTPPNTTPI, this peptide has 15 length amino acids (i.e., the sequence window width is 15) and the central amino acid T is phosphorylated. Therefore, after obtaining the blasted pairs of aligned sequences, we then compare every two sequence windows based on two kinds of scores: sequence window similarity score (WS) and BLOSUM50 score.^50^ The first score considers whether the two sequence windows have the same central amino acids (CAAs) and whether the two amino acids aligned in the same position are same. Finally, we defined the number of the same amino acids as WS the using the following formulas:

(Equation 2) $${WS}_{i}=\left\{ \begin{array}{cc} 1, & if\;A_{query\;i}=A_{ref\;i}\\ 0, & if\;A_{query\;i}\neq A_{ref\;i} \end{array}\right.$$

(Equation 3) $$\text{WS}=\sum_{i}^{w.width}{WS}_{i}$$

Where $A_{query\;i}$ means the ith amino acids in the query peptide sequence, $A_{ref\;i}$ means the ith amino acids in the reference peptide sequence, and *w.width* indicates the sequence window width (See examples in Figure S1B). The second score is built using blocks of aligned sequences that had no more than 50% identity and implemented with the pairwiseAlignment function in Biostrings package.

In addition, the CAA matching degree, the sequence window width, the threshold of the sequence window similarity score and the BLOSUM50 score can be adjusted by users in *PTMoreR*.

#### Motif enrichment analysis and kinase-substrate annotation

The motif enrichment analysis was implemented with an iterative statistical approach.^9^^,^^111^ Motifs with E-value <0.01 were considered to be significantly enriched. To facilitate motif visualization, the ggseqlogo package^101^ was incorporated in *PTMoreR*. Additionally, the kinase-substrate annotation information was sourced from the PhosphoSitePlus database.^53^ Based on the annotation results, users can obtain three tables: (i) The annotated kinase-substrate pair table containing the relevant UniProt IDs, gene names, phosphorylation peptides and sites information; (ii) The node table and (iii) the edge table, in which the annotated kinases/substrates are nodes and the relationships between the kinases and the substrates are edges. Furthermore, the annotation network plot was accomplished using ggraph package.

#### Data collection of phosphoproteomes in 11 mammals

The previous published dataset in which on average 12,400 P-sites in skin-derived fibroblast cells across 11 common mammalian species by phosphoproteomic DIA-MS (or Phos-DIA) were downloaded from PRIDE PXD028979.^15^ Briefly, this dataset was generated from human skin fibroblast (SF) cells purchased from American Type Culture Collection (CRL-4001), as well as *B. taurus* (cow), *C. lupus* (dog), *E. caballus* (horse), *F. catus* (cat), *M. mulatta* (monkey), *M. domestica* (opossum), *O. cuniculus* (rabbit), *O. aries* (sheep), *R. norvegicus* (rat), and *S. scrofa* (pig) SFs obtained from fresh skin tissue following established protocols.^15^

#### Protein-protein relationship visualization with the PTM sites regulation

For further presenting PTM sites expression visualization based on a PPI database (e.g., SARS-CoV-2 virus-Human PPI database^112^) or any protein ∼ protein relationships as measured by e.g., protein correlation profiling (PCP) approaches. Herein, users need to upload a PTM site level quantitative matrix that can be obtained from many commonly used software tools (MaxQuant,^48^ Proteome Discoverer^113^ and Spectronaut^49^). Users can additionally upload a user-defined PPI database (for example, in the case study on the phosphorylation landscape of SARS-CoV-2 infection, we prepared the phosphorylation sites quantification table from the reported phosphoproteomics dataset of SARS-CoV-2 infection in Vero E6 cells^68^^,^^114^ as well as the SARS-CoV-2 virus-host protein-protein interaction data including 32 human proteins interacting with 27 (26 wild-type and 1 mutant) viral proteins.^112^ Next, *PTMoreR* used the median value of every sample by default to normalize PTM site values and then performed a log2 transformation.^110^ In addition, those sites with over 50% missing values across all the samples were removed and then missing values were imputed with the k-Nearest Neighbor algorithm provided in NAguideR.^115^^,^^116^ Finally, the interaction visualization was implemented with the igraph package.^117^

#### Software implementation

*PTMoreR* is a web-based platform that built in R (Version 4.1.1, https://www.r-project.org/),^118^ and the GUI was implemented using R Shiny framework (version 1.6.0, https://github.com/rstudio/shiny).^119^ The online version was deployed on an in-house server with an Ubuntu Linux system according to the Shiny server professional administrator’s guide and can be accessed freely without any login requirement. It is platform independent and is fully compatible with many common browsers (Google Chrome, Mozilla Firefox, Safari, etc.). Furthermore, users can also operate this tool locally by a simple command "PTMoreR::PTMoreR_app()" in R after installation. The source codes of *PTMoreR* are also available at https://doi.org/10.5281/zenodo.10077642 and in the GitHub repository: https://github.com/wangshisheng/PTMoreR under an MIT license supporting for local installation. For detailed instructions on using this tool, please refer to the step-by-step manual available at https://doi.org/10.5281/zenodo.10077642 and in https://github.com/wangshisheng/PTMoreR/blob/master/manual.pdf.

### Quantification and statistical analysis

The phylogenetic tree with annotation data was implemented by ggtree package v3.6.0 and itol.toolkit package v1.1.7.^99^^,^^100^ The pairwise distances of the clustering trees were calculated by ape package v5.6.2.^102^ The heatmaps were visualized by pheatmap package v1.0.12. Sequence comparison analysis was conducted by IceLogo (https://iomics.ugent.be/icelogoserver).^103^ Kinase family tree was depicted by Coral (http://phanstiel-lab.med.unc.edu/CORAL/).^104^ The complex network plots were implemented by Cytoscape v3.9.1.^105^ The statistical significance was tested by samr package v3.0 with 1000 permutations at an FDR threshold of 0.01.^66^ Differentially expressed Phospho-peptides/sites were identified with FDR <0.01 and the absolute value of logarithmic fold changes with base 2 (|Log2(FCs)|) > 1 (i.e., a relative fold change of 2-folds). The *p* values between two categories were calculated by Wilcoxon rank-sum test and those among three or more categories were accomplished by Kruskal-Wallis rank-sum test. The kinase-substrate enrichment analysis was performed using Fisher’s exact test (fisher.test function with defaults in R). *p* values were corrected for multiple testing with the Benjamini–Hochberg (BH) method (p.adjust function with method “BH” in R). Gene Ontology (GO) enrichment analysis was performed by clusterProfiler package v4.6.0.^106^

## Supplemental information

Document S1. Figures S1–S7 and Table S1

Table S2. The 115,179 and 42,997 phosphorylation sites (P-sites) for human and mouse phosphoproteomes obtained from two recent large-scale studies, related to Figure 2 Two sheet names here are marked as “Human.identified” and “Mouse.identified” respectively.

Table S3. The information and organism identifiers for the 129 mammalian species downloaded from UniProt (http://www.uniprot.org/), related to Figure 3

Table S4. The features and the detectability matrix of P-sites in the Human.identified phosphoproteome data across species, related to Figures 2 and 4 Column 1 to 3 are UniProt ID, residue, and P-site position, respectively. Column 4 to 17 are the annotated phosphoproteome features that used in the Figure 2C, Figures 4B, 4E, 4F. Column 18 to 146 are the detectability matrix, in which the values are 0 for those sites not mapped in corresponding species and 1 for those mapped. The window similarity scores are ≥ 8.

Table S5. The GO terms with the BH-adjusted *p* < 0.01 in one of the Q1–Q5 segments and *p* > 0.05 in the other four from the GO enrichment analysis based on corresponding phosphoproteins, related to Figure 4

Table S6. The results with BH-adjusted *p* < 0.01 from the kinase enrichment analysis in each of the Q1–Q5 segments, related to Figure 5

Table S7. The phosphorylation expression data and statistical results from 10 common mammalian species (EAOG [i.e., human, rat, rabbit, monkey] and LAUT [i.e., cow, horse, pig, dog, cat, sheep]), related to Figure 6

Document S2. Article plus supplemental information
