# Supplementary material for: PTMoreR-enabled cross-species PTM mapping and comparative phosphoproteomics across mammals
Source: Cell Rep Methods. 2024 Sep 9;4(9):100859. doi: 10.1016/j.crmeth.2024.100859 (PMC11440062; doi:10.1016/j.crmeth.2024.100859)
Supplement: Document S1. Figures S1–S7 and Table S1 [file mmc1.pdf]

Cell Reports Methods, Volume *4*

Supplemental information

PTMoreR-enabled cross-species PTM mapping and comparative phosphoproteomics across mammals

Shisheng Wang, Yi Di, Yin Yang, Barbora Salovska, Wenxue Li, Liqiang Hu, Jiahui Yin, Wenguang Shao, Dong Zhou, Jingqiu Cheng, Dan Liu, Hao Yang, and Yansheng Liu

Supplemental information

PTMoreR-enabled cross-species PTM mapping and comparative phosphoproteomics across mammals

Shisheng Wang, Yi Di, Yin Yang, Barbora Salovska, Wenxue Li, Liqiang Hu, Jiahui Yin, Wenguang Shao, Dong Zhou, Jingqiu Cheng, Dan Liu, Hao Yang, and Yansheng Liu

I. Supplementary Figures

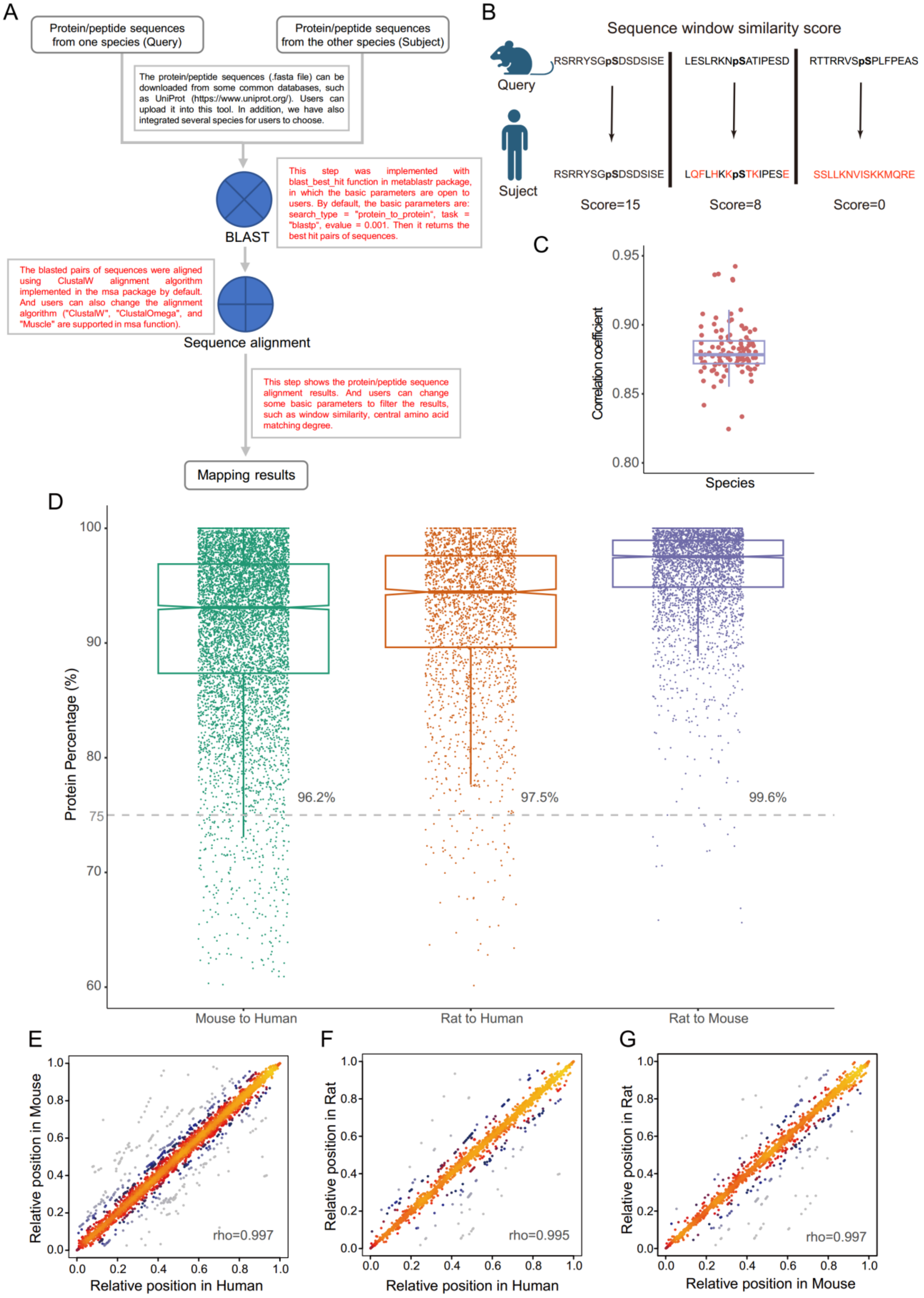

Figure S1. **Overview of the PTMoreR alignment functions and the blasting results of *PTMoreR* by cross mapping the Human, Mouse and Rat phosphoproteomes based on the PhosphoSitePlus database. Related to Figure 1. A.** Workflow of protein/peptide sequence BLAST and alignment between any two species. The steps in red words denotes parameters open to the users for them to process their data. The detailed implementation in *PTMoreR* can be found in Supplementary Notes. **B.** Three examples for the calculation of the sequence window similarity score. The peptide sequence window width is 15. If the amino acids are all same from a query sequence to a subject sequence, the score is 15 (see the left example). If some amino acids are mismatched (see the orange marked amino acids in the middle example) and their contribution scores are 0, the final score is 8. If none of the amino acids match correctly, the score is 0 (see the right example). **C.** Distribution of the correlation coefficient between the sequence window similarity score and the amino acid similarity BLOSUM50 score for each species. **D.** Distributions of the percentage of protein sequences blasted between Mouse, Rat, and Human species. **E-G**. Scatterplots of the relative position of the phosphosite between Mouse, Rat, and Human species.

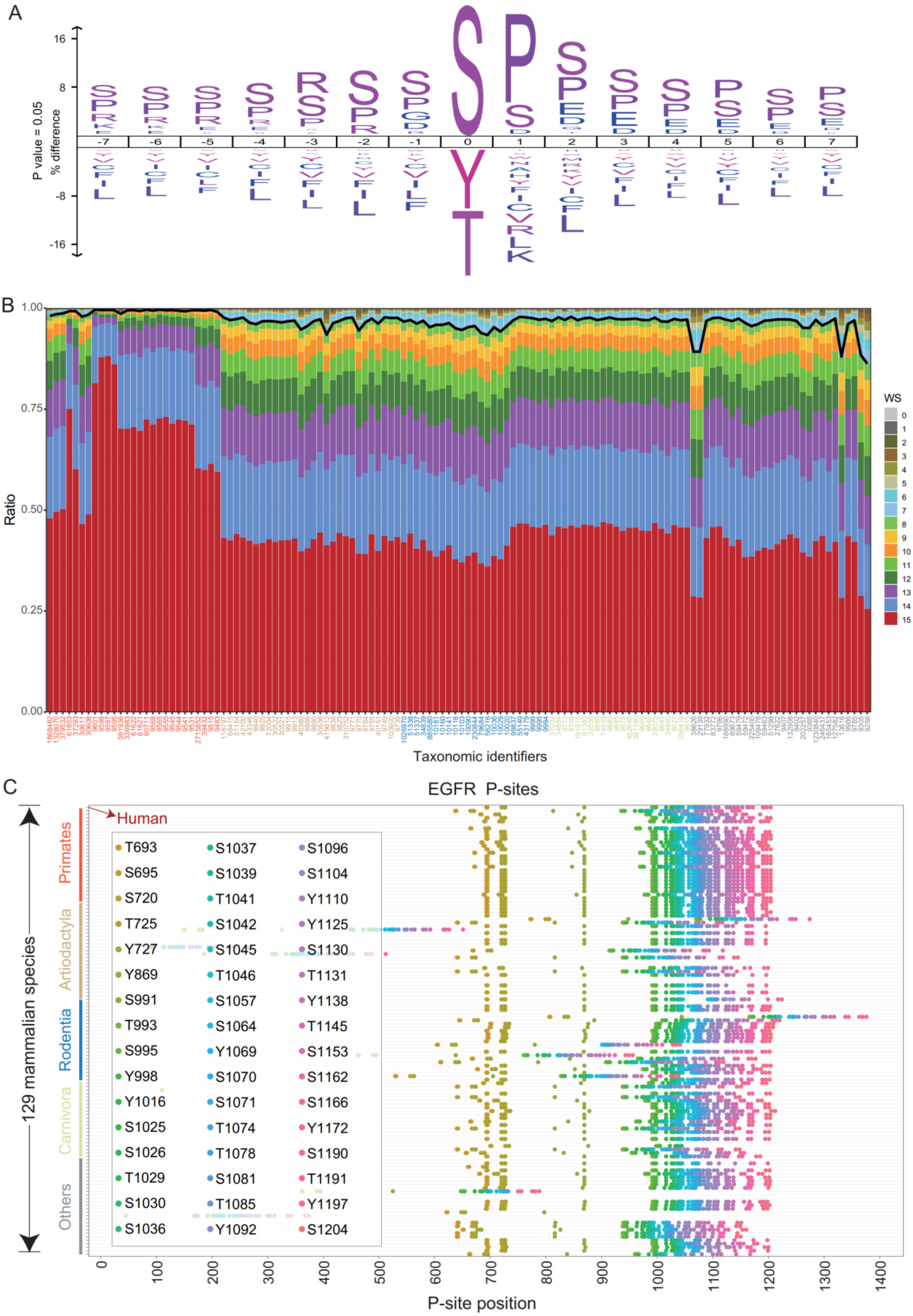
A
P value = 0.05
% difference
16
8
-8
-16
-7
-6
-5
-4
-3
-2
-1
0
1
2
3
4
5
6
7
B
1.00
0.75
0.50
0.25
0.00
Ratio
Taxonomic identifiers
WS
0
1
2
3
4
5
6
7
8
9
10
11
12
13
14
15
C
EGFR P-sites
Human
129 mammalian species
Primates
Artiodactyla
Rodentia
Carnivora
Others
T693
S695
S720
T725
Y727
Y869
S991
T993
S995
Y998
Y1016
S1025
S1026
T1029
S1030
S1036
S1037
S1039
T1041
S1042
S1045
T1046
S1057
S1064
Y1069
S1070
S1071
T1074
T1078
S1081
T1085
Y1092
S1096
S1104
Y1110
Y1125
S1130
T1131
Y1138
T1145
S1153
S1162
S1166
Y1172
S1190
T1191
Y1197
S1204
0
100
200
300
400
500
600
700
800
900
1000
1100
1200
1300
1400
P-site position

Figure S2. **The cross-species mapping of P-sites based on human phosphoproteome, related to Figure 2. A.** Sequence analysis of the flanking amino acids (±7 amino acids) around the pS, pT and pY (the Human.identified sequences versus the Theoretical.Blasted sequences). The percentage of significant residues (*P* value < 0.05) were shown. **B.** Stacked barplot showing the distribution of the ratio of every sequence window similarity score (0-15) based on phosphopeptides mapping from each of 128 mammalian species protein sequences to human sequences. "WS" means window similarity. The black line shows the cumulative ratio of WS >= 8. **C.** Distribution of EGFR P-site positions among 129 mammalian species. The P-site positions from Human are shown as reference in the top one.

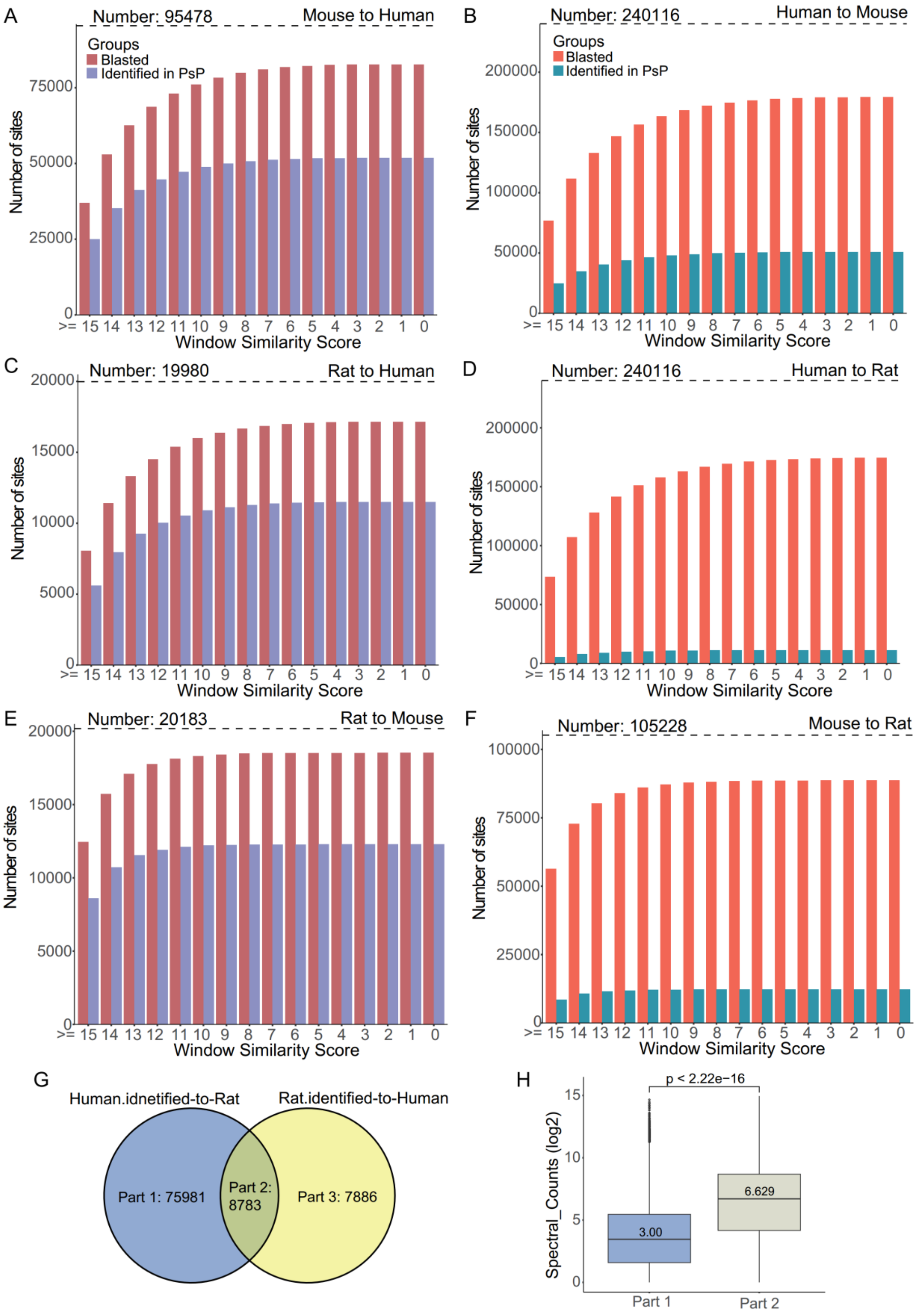
A
Number: 95478
Mouse to Human
Groups
Blasted
Identified in PsP
Number of sites
Window Similarity Score
B
Number: 240116
Human to Mouse
Groups
Blasted
Identified in PsP
Number of sites
Window Similarity Score
C
Number: 19980
Rat to Human
Number of sites
Window Similarity Score
D
Number: 240116
Human to Rat
Number of sites
Window Similarity Score
E
Number: 20183
Rat to Mouse
Number of sites
Window Similarity Score
F
Number: 105228
Mouse to Rat
Number of sites
Window Similarity Score
G
Human.idnetified-to-Rat
Rat.identified-to-Human
Part 1: 75981
Part 2: 8783
Part 3: 7886
H
p < 2.22e−16
Spectral_Counts (log2)
3.00
6.629
Part 1
Part 2

Figure S3. The functional annotation performance of *PTMoreR* by cross mapping the Human, Mouse and Rat phosphoproteomes based on the PhosphoSitePlus database, related to Figure 2. **A-F.** The cumulative distribution of the Mouse-to-Human (A), Human-to-Mouse (B), Rat-to-Human (C), Human-to-Rat (D), Rat-to-Mouse (E) and Mouse-to-Rat (F) sequence window similarity scores, respectively. **G.** The overlaps between the P-sites from Human.identified-to-Rat and those from Rat.identified-to-Human. **H.** The boxplots of the "Spectral_Counts (log2)" from the 75,981 P-sites and the 8,783 P-sites (accounting for 52.69% of the assembled rat dataset in the PhosphoSitePlus database) shown in the Venn plot. "Spectral_Counts (log2)" data were obtained from the comprehensive human phosphoproteome study.

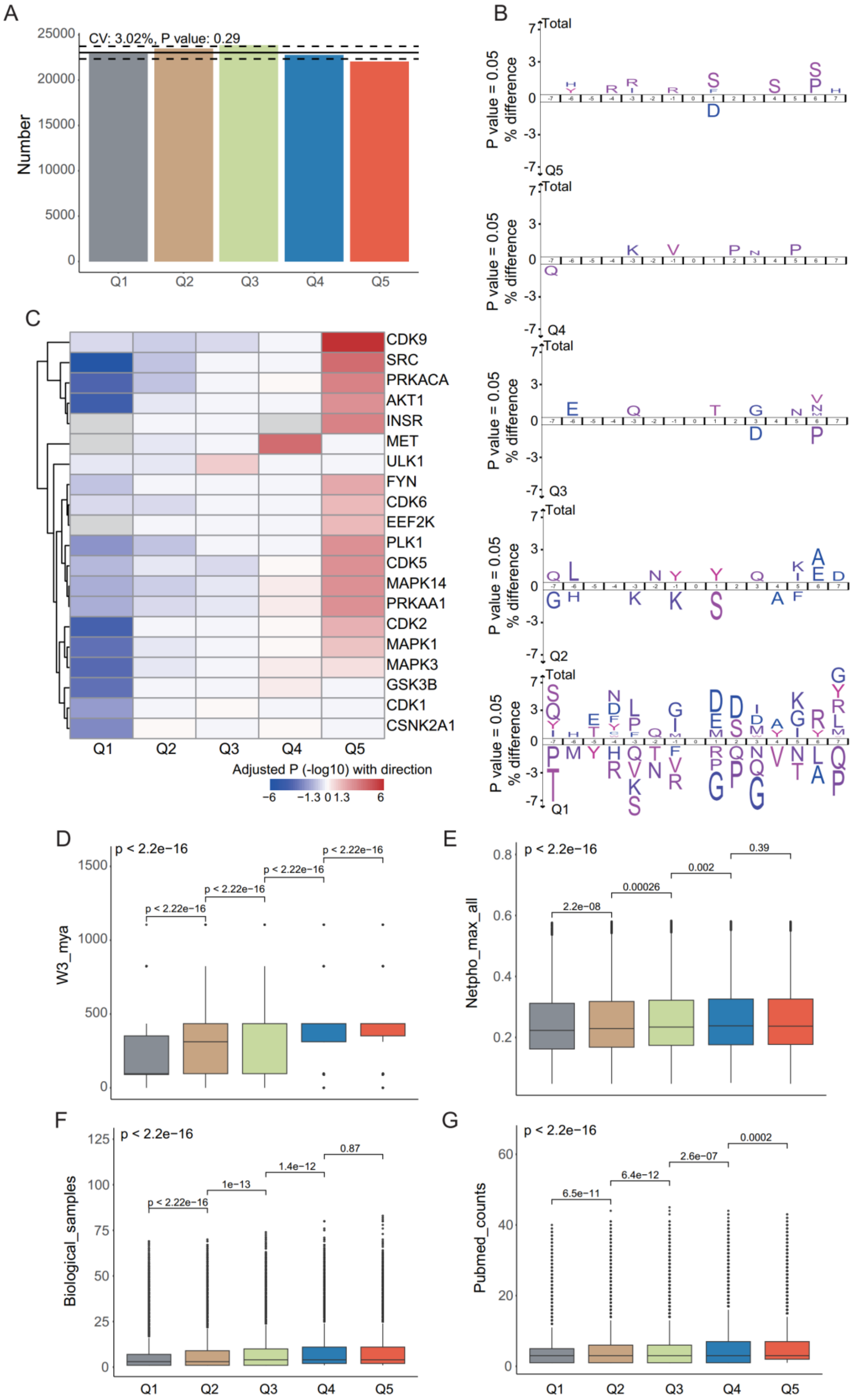
A
CV: 3.02%, P value: 0.29
Number
25000
20000
15000
10000
5000
0
Q1
Q2
Q3
Q4
Q5
B
Total
Q5
Q4
Q3
Q2
Q1
P value = 0.05
% difference
7
3
0
-3
-7
C
CDK9
SRC
PRKACA
AKT1
INSR
MET
ULK1
FYN
CDK6
EEF2K
PLK1
CDK5
MAPK14
PRKAA1
CDK2
MAPK1
MAPK3
GSK3B
CDK1
CSNK2A1
Adjusted P (-log10) with direction
−6
−1.3 0 1.3
6
D
W3_mya
p < 2.2e−16
p < 2.22e−16
E
Netpho_max_all
p < 2.2e−16
2.2e−08
0.00026
0.002
0.39
F
Biological_samples
p < 2.2e−16
p < 2.22e−16
1e−13
1.4e−12
0.87
G
Pubmed_counts
p < 2.2e−16
6.5e−11
6.4e−12
2.6e−07
0.0002

Figure S4. **P-site segmentation-based conservation analysis for amino acid distribution around pY and distribution of "W3_mya", "Netpho_max_all", "Biological_samples", and "Pubmed_counts" scores from Q1 to Q5. Related to Figure 3 and 4. A.** Distribution of the P-sites number in each segment. "CV" means coefficient of variation. P value was obtained from Grubbs test. **B.** Sequence analysis of the flanking amino acids (±7 amino acids) around the pY (each segment versus total identified sequences). The percentage of significant residues (*P* value < 0.05) were shown. **C.** Heatmap visualizing the significantly enriched kinases in any segment (P-site number >= 8 and BH-adjusted *P* value < 0.05, Fisher's exact test). **D-G.** W3_mya: Age of inferred ancestral species containing the site based on window of 3 residues. Netpho_max_all: Max Netphorest match for all models. Biological_samples: Number of biological samples in which site was detected by MS. Pubmed_counts: Number of publicly available quantitative studies reporting the site. P values: Wilcoxon rank sum test between two segments, Kruskal-Wallis rank sum test among five segments.

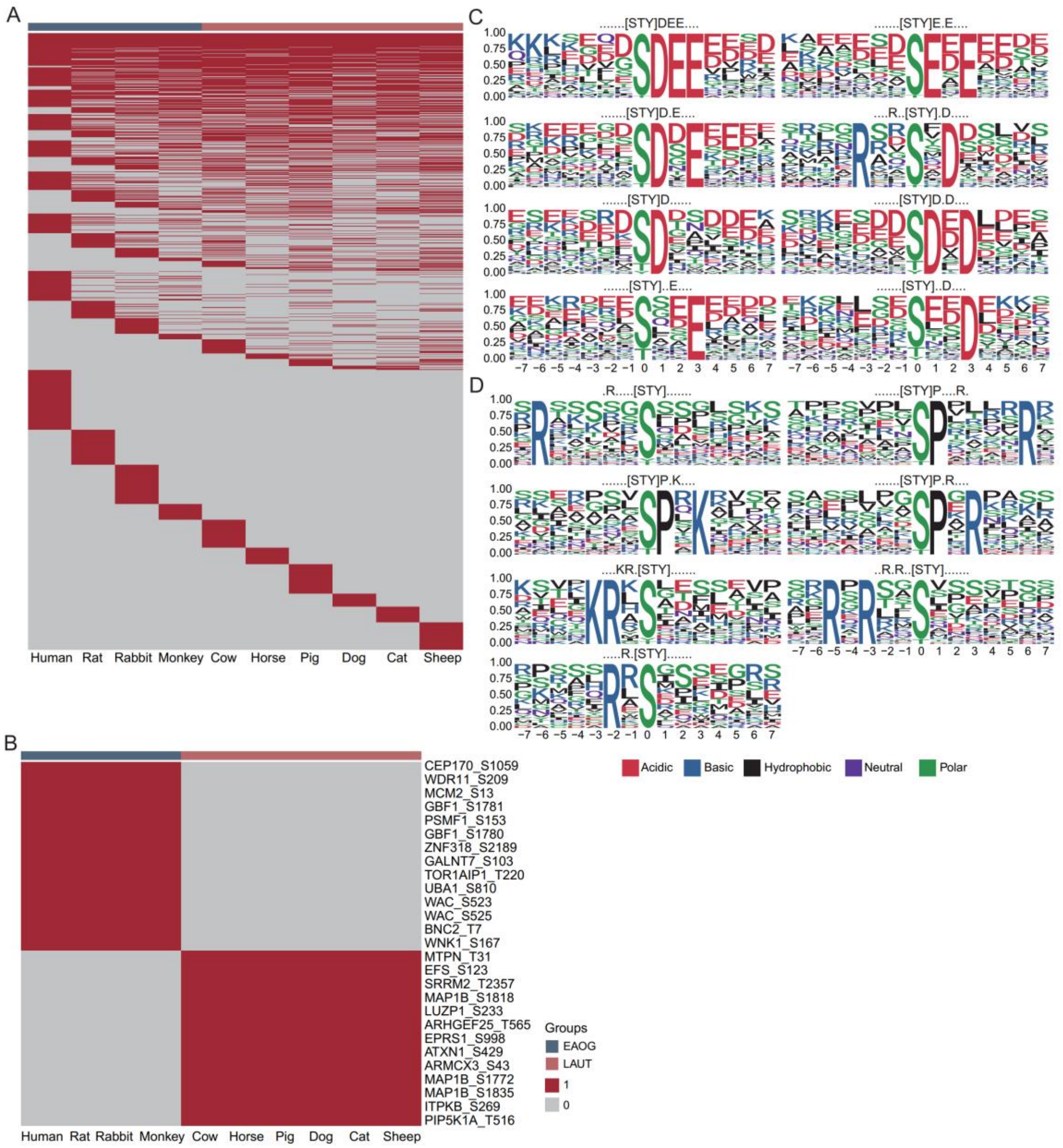

Figure S5. **Missing values after PTMoreR mapping for EAOG and LAUT species and the example motif enrichment analysis of the SARS-CoV-2-host protein-protein interactions in Vero E6 cells. Related to Figure 6 and 7. A.** Heatmap visualizing all P-sites that identified in EAOG and LAUT. **B.** Heatmap visualizing the P-sites that only existed in EAOG and LAUT. “1” means existed ones using dark red color, “0” means missing ones using the grey color. **C.** Motif plots of the up-regulated phosphopeptides across six time points (0, 2, 4, 8, 12, and 24 h) obtained from the Mfuzz analysis. **D.** Motif plots for the down-regulated phosphopeptides.

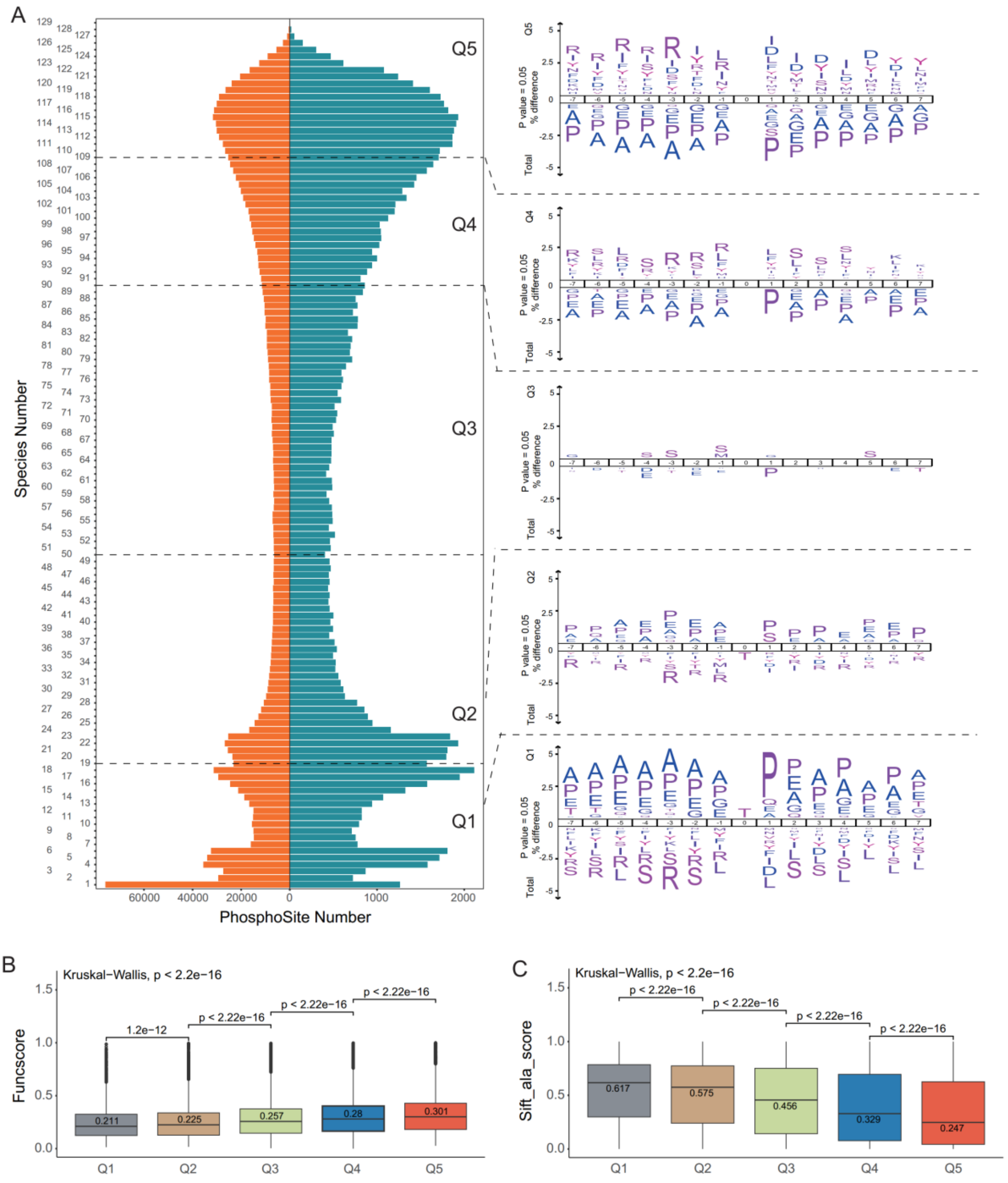

Figure S6. **Cross-species co-expression analysis among the 129 mammalian species using the window similarity score >= 14, related to Figure 4. A.** Left parts: Distributions of the number of the theoretical and the identified P-sites existed in the 129 mammals, which were divided into five segments (Q1-Q5) with a similar number of P-sites. "Blasted" means those theoretical P-sites in each species blasted to Human, and "Human.identified" means those "Blasted" P-sites identified in the identified P-sites (based on Human.identified). The window similarity scores here are >= 14. Right parts: Sequence analysis of the flanking amino acids (±7 amino acids) around the pT and pS (each segment versus total Human.identified sequences). The percentage of significant residues (*P* value < 0.05) were shown. **B.** Distributions of the site-specific functional score and sift_ala_score of the P-sites in each segment. *P* values, Wilcoxon rank sum test between two segments, Kruskal-Wallis rank sum test among five segments.

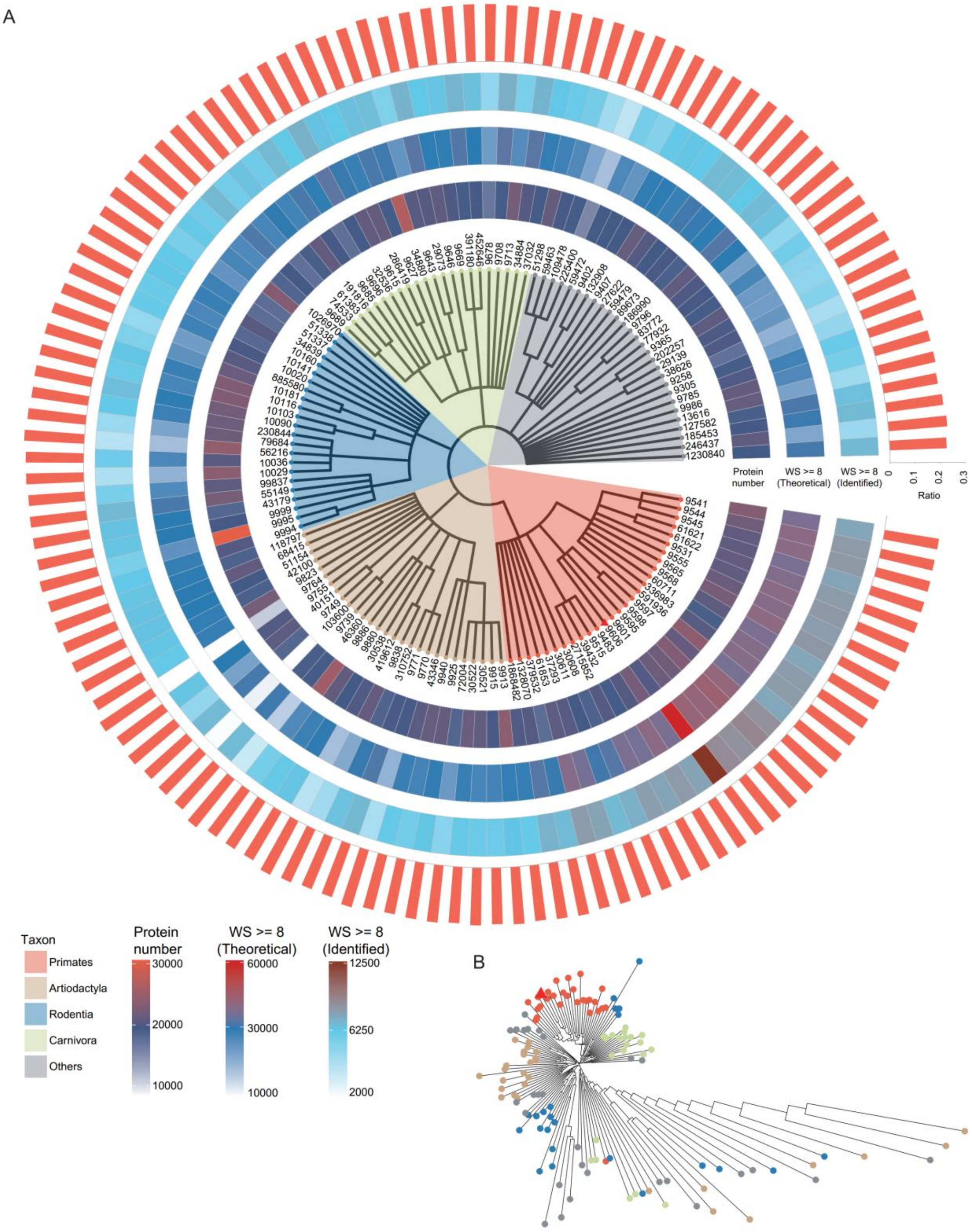

Figure S7. **Overview of phylogenetic N-glycosylation (N-X-S/T) mapping atlas across 129 mammalian species, related to Figure 3. A.** From inside to outside, circos plots visualizing the relationships, protein number, theoretical phospho-peptide number with WS scores >= 8, identified phospho-peptide number with WS scores >= 8, the ratios between the theoretical phospho-peptide number and the identified phospho-peptide number among the 129 mammalian species. "WS" means window similarity. **B.** The clustering trees based on the sparse matrix of glycosylation sites after PTMoreR mapping.

Table S1. Main features implemented in PTMoreR compared to other existing software/databases, related to Figure 1.

Features / Tool names	GUI	Pre-alignment	Specific sequence lookup	Motif-centric cross-species PTM mapping	Motif enrichment analysis	Motif similarity	KS annotation and enrichment analysis	PTM interaction Plot
**PTMap**	×	√	×	×	×	×	×	×
**PTMphinder**	×	√	×	×	√	×	×	×
**MoMo**	√	√	×	×	√	×	×	×
**MotifeR**	√	√	√	×	√	×	×	×
**iPTMnet**	√	√	×	×	×	×	×	×
**Phospho.ELM**	√	×	×	×	×	×	×	×
**DAPPLE**	√	√	×	×	×	×	×	×
**PhosphOrtholog**	√	√	×	×	×	×	×	×
**PhosphoBlast**	√	√	×	×	×	×	×	×
**PhosphoSitePlus**	√	×	×	×	×	×	×	×
**PTMoreR**	√	√	√	√	√	√	√	√

Symbols used for feature evaluations with "√" for present, "×" for absent.

Feature explanation for table S1:

1. "GUI" is short for graphical user interface to evaluate whether the software has a user-friendly interface.
2. "Pre-alignment" evaluates whether the software can align those uploaded peptide sequences with the background database (protein sequences) and force the modified sites/residues to be central sites.
3. "Specific sequence lookup" evaluates whether the software can check if the aligned peptides contain some specific sequences of users' interest (e.g., a known motif of a kinase).
4. "Motif-centric cross-species PTM mapping" evaluates whether the software support to process cross-species PTM mapping using motif information.
5. "Motif enrichment analysis" evaluates whether the software has the function to process motif enrichment analysis for standard peptides.
6. "Motif similarity" here means whether the software could evaluate how similar a motif PWM (position weight matrix) calculated from those human kinase substrates is to a motif PWM calculated from sequences in mammalian species corresponding to one particular human substrate.

7. “KS annotation and enrichment analysis” evaluates whether the software enable kinase-substrate annotation and site-specific functional enrichment analsis.

8. “PTM interaction Plot” evaluates whether the software is convenient for the users to display a network diagram of protein-protein-modification sites relationships. This function mainly shows the plot of the interaction between the uploaded and the blasted peptides/proteins.
